# Metabolic profiling and biological activity of two *Livistona* species: *L. chinensis* and *L. australis*†

**DOI:** 10.1039/d3ra01229h

**Published:** 2023-05-15

**Authors:** Seham S. El-hawary, Ahlam Elwekeel, Sara O. Abo El-Ela, Usama Ramadan Abdelmohsen, Asmaa I. Owis

**Affiliations:** a Department of Pharmacognosy, Faculty of Pharmacy, Cairo University Cairo Egypt; b Department of Pharmacognosy, Faculty of Pharmacy, Beni-Suef University Beni-Suef Egypt Ahlam_hasham2000@yahoo.com Ahlam.hassanain@pharm.bsu.edu.eg; c Department of Pharmacognosy, Faculty of Pharmacy, Minia University Minia Egypt; d Department of Pharmacognosy, Faculty of Pharmacy, Deraya University New Minia Egypt; e Department of Pharmacognosy, Faculty of Pharmacy, Heliopolis University for Sustainable Development Cairo Egypt

## Abstract

*Livistona* is a genus of family Arecaceae and widely grown in tropical areas. The phytochemical analysis of the leaves and fruits of two *Livistona* species, *L. chinensis* and *L. australis* were carried out using UPLC/MS and determination of the total phenolic and total flavonoid contents, in addition to the isolation and identification of five phenolic compounds and one fatty acid from *L. australis* fruits. The total phenolic compounds varied from 19.72 to 78.87 mg GAE g^−1^ dry plant, while the total flavonoid contents were in the range of 4.82–17.75 mg RE g^−1^ dry plant. The UPLC/MS analysis of the two species led to the characterization of forty-four metabolites belonging mainly to the different classes of flavonoids and phenolic acids, while the compounds isolated from *L. australis* fruits were identified as gallic acid, vanillic acid, protocatechuic acid, hyperoside, quercetin 3-*O*-α-d-arabinopyranoside and dodecanoic acid. The *in vitro* biological evaluation of *L. australis* leaves and fruits were estimated as anticholinesterase, telomerase reverse transcriptase (TERT) potentiation and anti-diabetic through measuring the capacity of the extracts to inhibit dipeptidyl peptidase (DPP-IV). The results revealed that the leaves showed remarkable anticholinesterase and antidiabetic activity compared to fruits with IC_50_ values of 65.55 ± 3.75 ng mL^−1^ and 90.8 ± 4.48 ng mL^−1^, respectively. In the TERT enzyme assay, the leaves extract triggered a 1.49-fold increase in telomerase activity. This work showed that the *Livistona* species are a good source for flavonoids and phenolics, which play an important role in anti-aging and the treatment of chronic diseases, such as diabetes and Alzheimer's.

## Abstract 2

*Livistona* extracts are rich sources of phenolics and used in managing chronic diseases.

## Graphical abstract

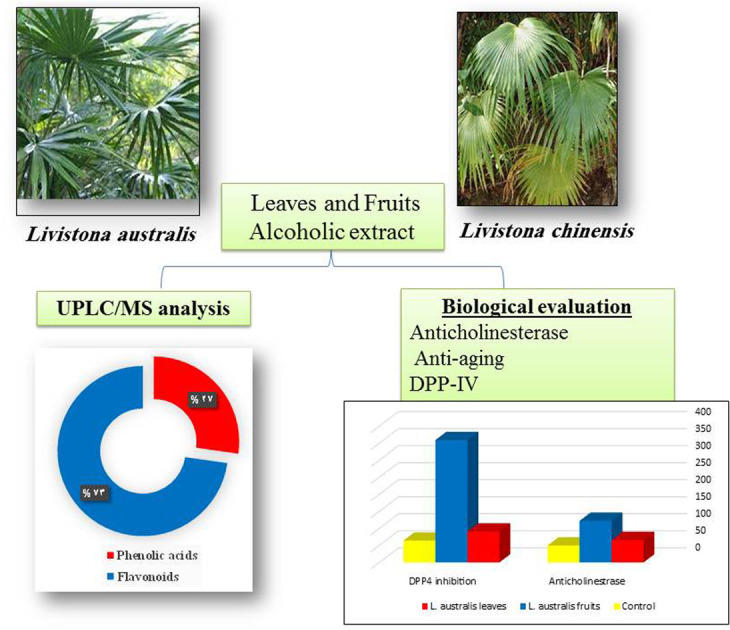

## 1. Introduction

Alzheimer's disease (AD) is a neurodegenerative disorder and is considered to be the main cause of dementia. Worldwide, about 40 million people are expected to suffer from dementia, and this number is expected to be doubled by 2050.^1^ A low level of acetylcholine in the brain is the key marker of the disease. Inhibition of the acetylcholinesterase (AChE) enzyme, which is responsible for the degradation of acetylcholine, is the most promising approach for the treatment of AD. Many natural compounds, such as terpenoids, coumarins, flavonoids, alkaloids and other phenolic compounds, have shown anticholinesterase activity.^2^

Another sign associated with aging is telomere attrition. At the ends of linear chromosomes are DNA repeats called telomeres, which ensure chromosome stability throughout replication. Every time a cell divides, as well as when there is oxidative stress, the telomere length is decreased. When telomeres significantly shorten in length, cells can no longer divide, causing cell death. There is an enzyme called telomerase, which creates the particular DNA sequence at the telomeres.^3–7^ The substances that potentiate telomerase are called telomerase activators and are used as antiageing sources.

Another heath problem that has an impact on human health is T2DM. The number of patients expected to suffer from diabetes in 2040 will be 642 million worldwide, according to the International Diabetes Federation.^8^ Lowering the blood glucose level is the primary treatment of T2DM, which can be done through α-glucosidase inhibitors or the stimulation of insulin secretion.^9^ Incretins are hormones that are released from the small intestine in response to dietary intake, and have an impact similar to glucagon. The main incretins are a glucagon-like peptide (GLP-1) and a glucose-dependent insulinotropic peptide (GIP). These incretins potentiate insulin secretion from pancreatic B-cells.^10^ Dipeptidyl peptidase-IV is responsible for the degradation of these incretins. Thus, DPP-IV inhibitors prevent the degradation of these incretins, which led to an increase in the insulin level and lowering of the blood glucose level.^11^

*Livistona* is a genus belonging to the family Arecaceae and it is widely distributed in tropical areas, with about 36 species reported in the genus. Phytochemical studies on *Livistona* have reported on many compounds, such as flavonoids, phenolics, ceramides and glycerides,^12–14^ pyranone derivatives^15^ and sulfated flavonoids.^16^ Biologically, *L. chinensis* has been reported to have anticancer^17^ and cardioprotective^18^ properties, while *L. decipiens* has been reported to have antioxidant and cytotoxic activities.^19^ A recent report on the leaves and fruit extracts of the plant also showed inhibitory activity against coronavirus.^20^ The fruits of *L. chinensis* are edible in eastern Asia, they are a component of prepared soups used in the treatment of liver cancer and chronic hepatitis.^21^ The nutritional evaluation of the leaves and fruits of *L. australis* also showed that the fruits are a good source of calcium and potassium.^22^ This work aims to identify the secondary metabolites in the leaves and fruits of *L. chinensis* and *L. australis* using UPLC coupled with high-resolution electron spray ionization mass (HRESIMS), and evaluate their anticholinesterase, DPP-IV inhibitory potential, and telomerase activation potential.

## 2. Experimental

### 2.1. Plant material

The fruits and leaves of *Livistona australis* and *Livistona chinensis* were supplied from El-Orman Botanical Garden, Giza, Egypt on Jan. 2018. The samples were authenticated by Eng. Trease Labib (El Orman Botanical Garden) and Dr Reem Samir Hamdy (Plant Taxonomy, Botany Department, Faculty of Science, Cairo University, Giza, Egypt). Voucher specimens (BUPD-71-2018 for *L. australis* and BUPD-73-2018 for *L. chinensis*) were kept in the Pharmacognosy Department, Faculty of Pharmacy, Beni-Suef University, Egypt. The plants were cleaned, dried and finely powdered for the study.

### 2.2. Chemicals and reagents

Solvents such as *n*-hexane, dichloromethane (CH_2_Cl_2_), ethyl acetate (EtOAc), ethanol (EtOH), and methanol (MeOH), were obtained from the El-Nasr Company for Pharmaceuticals and Chemicals, Egypt. Acetonitrile, methanol (HPLC grade) and DMSO were purchased from (Sigma-Aldrich, St. Louis, MO, USA). Acetyl-cholinesterase inhibitor screening kits (cat. # IACE-100) were purchased from BioAssay Systems (Hayward, CA, USA). Donepezil (Cat # sc-218265) was purchased from Santa Cruz Biotechnology (Texas, USA). TERT ELISA kit (cat. # K4187-100) was purchased from Biovision incorporated (Milpitas, CA, USA), The curcumin standard for the TERT assay was obtained from Sigma-Aldrich, Germany.

### 2.3. Apparatus

The NMR analysis for the isolated compounds was carried out using Bruker Avance III 400 MHz (Bruker, USA). LC/MS analysis of the extracts was done on an Acquity Ultra Performance Liquid Chromatography (UPLC) system connected with a Synapt G2 HDMS quadrupole time-of-flight hybrid mass spectrometer (Waters, USA). Spectrophotometric determination of the total phenolic and flavonoid contents was measured using a Jenway 6305 spectrophotometer.

### 2.4. Metabolomics study

Three grams of each sample were exhaustively defatted using *n*-hexane (3 × 15 mL). The marc was then extracted with 70% ethanol, filtered with Whatman filter paper, and evaporated under vacuum to afford the dry residue. One mg of each extract was dissolved in 1 mL methanol and analyzed using LC/MS, as reported by Owis *et al.*, 2020.^23^ Briefly, the samples were analyzed using a C-18 column (2.1 × 100 mm, 1.7 μm particle size; Waters, Milford, MA, USA). A binary solvent was used for separation; solvent A was 0.1% formic acid in water, while solvent B was acetonitrile, and a gradient elution from 0–100% of solvent B in 6 min with flow rate 0.3 mL min^−1^ was followed. Electron spray ionization mass spectrometry was used to detect the metabolites; the processed data set was subjected to molecular formula prediction, as well as peak identification. The positive and negative ionization mode data sets from the plant extract were dereplicated against the Dictionary of Natural Products (DNP) database.

### 2.5. Determination of total phenolics and total flavonoids contents

The fruits and leaves of *L. australis* and *L. chinensis* at 1 g each were extracted with 50 mL of 80% methanol for 2 h on an orbital shaker set at 200 rpm. Each mixture was separately centrifuged for 20 min, and the supernatants were decanted. The residues were re-extracted under the same conditions. Supernatants were separately combined. The volume was adjusted to 100 mL; these extracts were used for total phenolic and total flavonoid determination. The total phenolic content was assessed using Folin–Ciocalteu reagent as follows.^24^ A volume of 300 μL of each extract was mixed with 2.25 mL Folin reagent. After 5 minutes, 2.25 mL of 6% sodium carbonate was added. The mixture was rested for 90 min, and the blue color that developed was measured at 725 nm. The results were expressed as mg gallic acid equivalents per g dried sample (mg GAE g^−1^). Meanwhile, the flavonoid content was assessed by colorimetric method.^24^ Briefly, 0.5 mL of each extract was diluted with 2.25 mL distilled water, and then 150 μL of 5% NaNO_2_ was added. Six min later, 300 μL of 10% AlCl_3_ was mixed and the reaction was rested for 6 min. Finally, 1 mL of 1 M NaOH was added. The mixture was mixed well and the absorbance was measured at 510 nm. Results of the total flavonoids were expressed as mg rutin equivalents per g of dried sample (mg RE g^−1^).

### 2.6. Extraction and fractionation

Air-dried, powdered pericarp of *L. australis* (1.75 kg) was extracted with 70% ethanol (5 L) by cold maceration for 72 h, and then filtered through filter paper. The marc was re-extracted again until exhaustion, and the collected extracts was dried under vacuum at 45 °C to afford the crude extract (340 g). A part of the dried extract (200 g) was fractionated using *n*-hexane, dichloromethane and ethyl acetate to give 22, 14 and 6 g, respectively. The *n*-hexane fraction (6 g) was chromatographed on a silica gel (E-Merck, Germany) column using hexane with 10% ethyl acetate increments to afford one major compound 1 (500 mg). The ethyl acetate fraction (5 g) was chromatographed on a polyamide (E-Merck, Germany) column starting with 100% water as the eluent, then with decreasing polarity by 20% MeOH to afford five sub-fractions. The sub-fraction eluted with 20% water (500 mg) was re-chromatographed on a Sephadex LH-20 column using methanol to afford compounds 2 and 3 (25, 10 mg, respectively). The sub-fraction eluted with 60% water (100 mg) was re-chromatographed on a Sephadex LH-20 column (Sigma-Aldrich, USA) using methanol as an eluent to afford three compounds 4–6 (15, 12, 20 mg, respectively).

### 2.7. Biological studies

#### 2.7.1. Anticholinesterase activity

The anticholinesterase potential of *L. australis* leaves and fruits extracts, as well as donepezil standard, were measured utilizing Ellman's method.^25^ The method depends on the cleavage of acetylthiocholine substrate into thiocholine by the acetylcholinesterase enzyme. Thiocholine reacts with Ellman's reagent [5,5′-dithiobis(2-nitrobenzoic acid), known as DTNB], to give 2-nitrobenzoate-5-mercaptothiocholine and 5-thio-2-nitrobenzoate, which can be detected at 412 nm. The experiment was done according to the manufacturer's instructions in triplicate, and the IC_50_ values for the tested extracts were calculated as follows; a mixture of 25 μL of acetylthiocholine iodide (5 mM), 125 μL of DTNB (3 mM), 50 μL of buffer B (50 mM Tris–HCl, pH 8, 0.1% BSA) and 25 μL of each test extract solution at different concentrations were added. The mixture was then incubated for 10 min at 37 °C, then 25 μL of AChE (0.05 U mL^−1^) was added to start the reaction. A negative control (25% DMSO in MeOH) was used.

#### 2.7.2. Antiaging activity

*In vitro* assay to measure TERT in response to the tested extracts and curcumin standard was carried out using the human fibroblast cell line (HFB4), which is known as the normal human skin melanocyte cell line. The assay was carried out according to the method reported by Refaey, Abdelhamid *et al.* 2021.^26^ HFB4 was obtained from (VACSERA, Giza, Egypt) and cultured in RPMI-1640 media supplemented with 10% heat-inactivated FBS, 100 U mL^−1^ penicillin, and 100 U mL^−1^ streptomycin. In a six well plate, the cells were plated with a density of 2 × 10^5^ cells per well and incubated at 37 °C for 24 h in a humid atmosphere of 5% CO_2_. The tested extracts and curcumin (positive control) were added to the cells. After 24 h, the supernatant was collected and centrifuged for 20 min, which was then used to carry out the assay. The collected supernatants were added to precoated microtiter plates with a biotin-detection antibody specific to TERT, then streptavidin conjugated to horse-radish peroxidase (SABC) was added to each microplate well and incubated. The color of the solutions in the wells containing TERT, the antibody and enzyme-conjugated streptavidin will change after the addition of 3,3′,5,5′-tetramethylbenzidine (TMB) substrate solution. The stop solution was used to stop the enzyme–substrate reaction, and the color change at 450 nm was determined spectrophotometrically. The optical density of the samples was then compared to the standard curve in order to determine the TERT concentration in the samples.

#### 2.7.3. Antidiabetic activity

The antidiabetic potential of the leaves and fruits extract was assessed by measuring the ability of the extract to inhibit DPP-IV using the DPP-IV inhibition screening kit (Catalog Number KA3738, Abnova, Taiwan). The assay depends on the ability of DPP-IV to break down a fluorogenic substrate, Gly-Pro-Aminomethylcoumarin, and release products measured by fluorometer (*E*_m_/*E*_x_ = 360/460 nm). In the presence of a DPP-IV inhibitor, the cleavage will be inhibited. The procedure of the experiment was done according to the supplier instructions, and as reported by Kempegowda, Zameer *et al.* 2018.^27^ Different concentrations of the tested extracts were dissolved in DMSO. A volume of 10 μL of the sample solution was mixed with 30 μL assay buffer and 10 μL enzyme solution in a 96-well plate, then 50 μL of a diluted substrate solution was added to start the reaction and incubated at 37 °C for 30 min. Fluorescence with an excitation wavelength of 360 nm and an emission wavelength of 460 nm was monitored using a plate reader. The IC_50_ for the tested extracts and sitagliptin was calculated.

### 2.8. Statistical analysis

Data were presented as the mean ± standard deviation (SD). All analyses were done in triplicate. Statistical and graphical evaluations were done using Graph Pad Prism 7 and Microsoft Excel 2010.

## 3. Results and discussion

### 3.1. Metabolic analysis

**Fig. 1:**
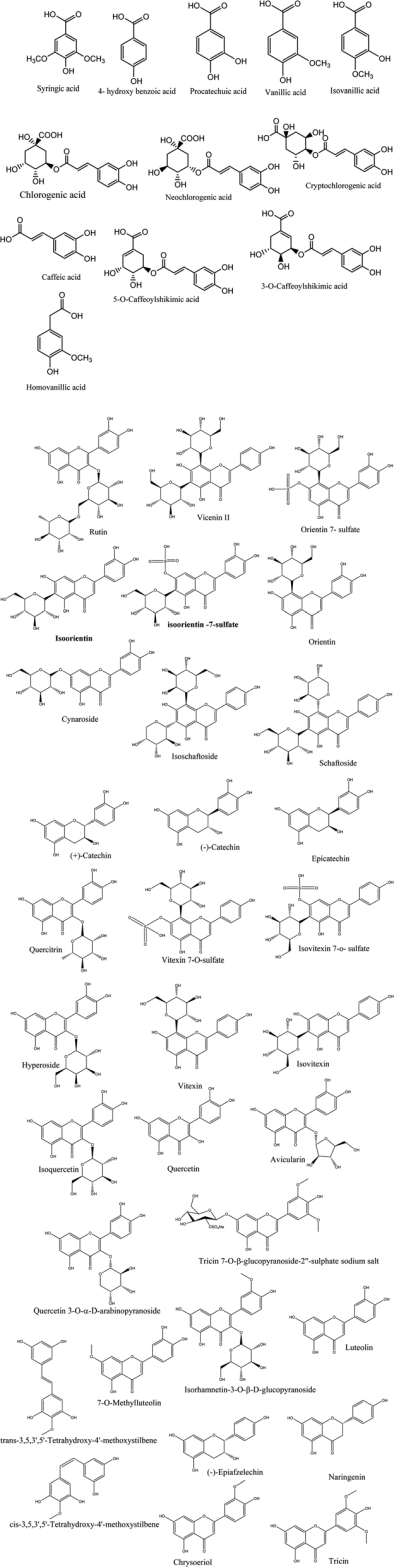
Structures of the dereplicated metabolites from the alcoholic extract of *L. chinensis* and *L. australis* leaves and fruits.

Metabolic profiling of the secondary metabolites in *L. chinensis* and *L. australis* leaves and fruits were conducted using LC-HRESIMS (chromatograms in ESI data, Fig. S1–S8†) for dereplication/detection of the putative compounds responsible for the various activities assigned to *Livistona*. It led to the characterization of forty-four compounds (Table 1, Fig. 1) belonging to different classes of flavonoids and phenolic acids.

**Table 1:** LC-HR-ESIMS dereplication results of the alcoholic extract of *L. chinensis* and *L. australis* leaves and fruits^a^

Metabolites name	*L. chinensis*		*L. australis*		MF	*m*/*z*
	L	F	L	F		
Syringic acid	+	+	+	+	C_9_H_10_O_5_	197.0447
4-Hydroxybenzoic acid	ND	+	ND	+	C_7_H_6_O_3_	137.0238
Protocatechuic acid	+	+	+	+	C_7_H_6_O_4_	153.0189
Rutin	ND	ND	ND	+	C_27_H_30_O_16_	609.1455
Chlorogenic acid	+	+	+	+	C_16_H_18_O_9_	353.0881
Cryptochlorogenic acid	+	+	+	+	C_16_H_18_O_9_	353.0881
Neochlorogenic acid	+	+	+	+	C_16_H_18_O_9_	353.0881
Vanillic acid	+	+	+	+	C_8_H_8_O_4_	167.0342
Isovanillic acid	+	+	+	+	C_8_H_8_O_4_	167.0342
Vicenin II	+	+	ND	ND	C_27_H_30_O_15_	593.1514
Isoorientin-7-*O*-sulfate	+	+	+	ND	C_21_H_20_O_14_S	527.0501
Orientin-7-*O*-sulfate	+	+	+	ND	C_21_H_20_O_14_S	527.0501
Orientin	+	+	+	+	C_21_H_20_O_11_	447.0931
Isoorientin	+	+	+	+	C_21_H_20_O_11_	447.0931
Cynaroside	+	+	+	+	C_21_H_20_O_11_	447.0932
Caffeic acid	+	+	+	+	C_9_H_8_O_4_	179.0346
5-*O*-Caffeoylshikimic acid	+	+	ND	+	C_16_H_16_O_8_	335.0769
3-*O*-Caffeoylshikimic acid	+	+	ND	+	C_16_H_16_O_8_	335.0769
Schaftoside	+	+	+	ND	C_26_H_28_O_14_	563.1405
Isoschaftoside	+	+	+	ND	C_26_H_28_O_14_	563.1405
(+)-Catechin	ND	+	+	+	C_15_H_14_O_6_	289.0713
(−)-Catechin	ND	+	+	+	C_15_H_14_O_6_	289.0713
Epicatechin	ND	+	+	+	C_15_H_14_O_6_	289.0713
Quercitrin	+	+	+	+	C_21_H_20_O_11_	447.0929
Isovitexin 7-*O*-sulfate	+	+	+	ND	C_21_H_20_O_13_S	511.0558
Vitexin 7-*O*-sulfate	+	+	+	ND	C_21_H_20_O_13_S	511.0558
Isovitexin	+	+	ND	ND	C_21_H_20_O_10_	431.0974
Vitexin	+	+	ND	ND	C_21_H_20_O_10_	431.0974
Hyperoside	ND	+	+	+	C_21_H_20_O_12_	463.0876
Isoquercetin	ND	+	+	+	C_21_H_20_O_12_	463.0876
Quercetin	ND	ND	ND	+	C_15_H_10_O_7_	301.0351
Avicularin	ND	ND	ND	+	C_20_H_18_O_11_	433.0977
Quercetin 3-*O*-α-d-arabinopyranoside	ND	ND	ND	+	C_20_H_18_O_11_	433.0977
Tricin 7-*O*-β-glucopyranoside-2′′-sulphate sodium salt	+	ND	+	ND	C_23_H_24_O_15_S	571.0759
Isorhamnetin-3-*O*-β-d-glucopyranoside	ND	+	ND	+	C_22_H_22_O_12_	477.1032
*cis*-3,5,3′,5′-Tetrahydroxy-4′-methoxystilbene	ND	+	ND	+	C_15_H_14_O_5_	273.0766
*trans*-3,5,3′,5′-Tetrahydroxy-4′-methoxystilbene	ND	+	ND	+	C_15_H_14_O_5_	273.0766
(−)-Epiafzelechin	ND	+	ND	+	C_15_H_14_O_5_	273.0766
Luteolin	+	+	+	+	C_15_H_10_O_6_	285.0395
Homovanillic acid	+	+	+	+	C_9_H_10_O_4_	181.0498
Naringenin	ND	+	ND	+	C_15_H_12_O_5_	271.0599
7-*O*-Methylluteolin	+	+	ND	+	C_16_H_12_O_6_	299.0558
Chrysoeriol	+	+	ND	+	C_16_H_12_O_6_	299.0558
Tricin	+	+	+	+	C_17_H_14_O_7_	329.0658

a L: leaves, F: fruits, MF: molecular formula, ND: not detected.

#### 3.1.1. Phenolic acids

Twelve phenolic acids (six hydroxybenzoic acids and six hydroxycinnamic acids) were annotated in the extracts of the leaves and fruits of *L. chinensis* and *L. australis*. The hydroxybenzoic acids were identified as syringic acid, 4-hydroxy benzoic acid, procatechuic acid, vanillic acid, isovanillic acid and homovanillic acid. They were characterized from the mass ion peaks at *m*/*z* 197.0447, 137.0238, 153.0189, 167.0342, 181.0498 and molecular formulas C_9_H_10_O_5,_ C_7_H_6_O_3,_ C_7_H_6_O_4_, C_8_H_8_O_4_ and C_9_H_10_O_4_, respectively. Syringic acid, procatechuic acid, vanillic acid, isovanillic acid and homovanillic were detected in the leaves and fruit extracts of *L. chinensis* and *L. australis*, while 4-hydroxy benzoic acid was detected only in the fruit extract of both plants. These phenolic acids were detected previously in *L. chinensis* leaves.^28^ A metabolite with a mass ion peak at *m*/*z* 353.0881 and molecular formula of C_16_H_18_O_9_ was detected in the leaves and fruit extracts of both plants, and was annotated as chlorogenic acid or its isomers cryptochlorogenic acid or neochlorogenic acid, which were previously detected in *L. chinensis* leaves.^28^ Also caffeic acid was recognized in all analyzed extracts from the mass ion peak at *m*/*z* 179.0346 and the molecular formula C_9_H_8_O_4_. 5-*O*-caffeoylshikimic acid or 3-*O*-caffeoylshikimic acid was characterized from the mass ion peak at *m*/*z* 335.0769 and molecular formula C_16_H_16_O_8_ in the fruit and leaves of *L. chinensis* and in the fruit extract of *L. australis*. Caffeic acid and caffeoylshikimic acid were previously reported in *L. chinensis* fruits.^29^

#### 3.1.2. Flavonoids

Thirty-two flavonoids were characterized in the leaves and fruit extracts of *L. chinensis* and *L. australis*. The metabolite with the mass ion peak at *m*/*z* 609.1455, in accordance with the molecular formula C_27_H_30_O_16_, was detected in the *L. australis* fruit extract and recognized as rutin, which was formerly isolated from the *L. chinensis* fruit extract.^30^ Another metabolite was detected in the *L. chinensis* leaves and fruit with molecular ion peak at *m*/*z* 593.1514, which is in agreement with the molecular formula C_27_H_30_O_15_ and was annotated as vienin II. This was previously reported in *L. decipiens* leaves.^19^ Moreover, the metabolites with the mass ion peaks at *m*/*z* 527.0501 and 511.0558 with the suggested molecular formulas C_21_H_20_O_14_S and C_21_H_20_O_13_S were identified as orientin-7-*O*-sulfate (or its isomer isoorientin-7-*O*-sulfate) and vitexin 7-*O*-sulfate (or its isomer isovitexin 7-*O*-sulfate), respectively. These metabolites were detected in *L. chinensis* leaves and fruits and *L. australis* leaves, and were previously reported in *L. chinensis* leaves.^28^ The mass ion peaks at *m*/*z* 447.0931 and 431.0974 with molecular formulas of C_21_H_20_O_11_ and C_21_H_20_O_10_ were identified as orientin (or its isomer isoorientin) and vitexin (or isovitexin), which are C-glycosyl flavones. Orientin was detected in all tested extracts, while vitexin was detected in *L. chinensis* leaves and fruits. Both compounds were previously reported in *L. chinensis* leaves.^18^ Another metabolite was detected in the leaves and fruits of *L. chinensis* and *L. australis* with a molecular ion peak at *m*/*z* 447.0932, and was in agreement with the molecular formula C_21_H_20_O_11_. It was identified as cynaroside (luteolin 7-glucoside) and previously reported in *L. chinensis* leaves.^28^ The metabolite with mass ion peak at *m*/*z* 563.1405 in agreement with the molecular formula C_26_H_28_O_14_ was identified as schaftoside (or isoschaftoside). The metabolite was detected in the leaves and fruits of *L. chinensis* and *L. australis* leaves, and was previously reported in *L. chinensis* leaves.^28^ The metabolite with mass ion peak at *m*/*z* 289.0713 in accordance with the molecular formula C_15_H_14_O_6_ was identified as catechin (flavan-3-ol). The compound was detected in the leaves and fruits of *L. australis* and *L. chinensis* fruits. It was previously reported in *L. chinensis* fruits.^30^ The metabolites with mass ion peaks at *m*/*z* 447.0929, 463.0876, 301.0351 and 433.0977 in accordance with the molecular formulas C_21_H_20_O_11_, C_21_H_20_O_12,_ C_15_H_10_O_7_ and C_20_H_18_O_11_ were characterized as quercitrin, hyperoside (quercetin 3-galactoside), isoquercitrin, quercetin and quercetin 3-arabinoside, respectively. Quercitrin was detected in all tested extracts, and previously reported in *L. chinensis* fruits.^30^ Meanwhile, hyperoside and isoquercitrin were detected in *L. chinensis* fruits, *L. australis* leaves and fruits, they were previously reported in *L. australis* leaves.^16^ Quercetin and quercetin 3-arabinoside were detected in *L. australis* fruits and previously reported in *L. australis* leaves and *Serenoa repens* fruit, respectively.^16,31^ The metabolite with mass ion peak at 571.0759 in agreement with the molecular formula C_23_H_24_O_15_S was identified as tricin 7-*O*-β-glucopyranoside-2′′-sulphate sodium salt, which was detected in *L. chinensis* and *L. australis* leaves and previously reported in *L. australis* leaves.^16^ The mass ion peak at *m*/*z* 477.1032 and molecular formula C_22_H_22_O_12_ was detected in the fruits of *L. chinensis* and *L. australis*, identified as isorhamnetin-3-*O*-β-d-glucopyranoside, and was previously reported in *L. chinensis* fruits.^30^ The metabolite with mass ion peak at *m*/*z* 273.0766 and molecular formula C_15_H_14_O_5_ was characterized as 3,5,3′,5′-tetrahydroxy-4′-methoxystilbene or (−)-epiafzelechin. The metabolite was detected in the fruit extracts of both species, and previously reported in *L. chinensis* fruits.^13^ The metabolites with mass ion peaks at 285.0395, 271.0599, 299.0558 and 329.0658 in agreement with the molecular formulas C_15_H_10_O_6_, C_15_H_12_O_5_, C_16_H_12_O_6_ and C_17_H_14_O_7_ were identified as luteolin, naringenin, 7-*O*-methylluteolin, and tricin, respectively. Luteolin and tricin were detected in all analyzed extracts, while naringenin was detected in the fruit extracts of both species. 7-*O*-Methylluteolin was detected in the leaves and fruits of *L. chinensis* and *L. australis* fruits. Luteolin and naringenin were previously reported in *L. australis* leaves, while 7-*O*-methylluteolin and tricin were reported in *L. chinensis*.^16,18,28,32^

### 3.2. Total phenolic and total flavonoids content

Folin–Ciocalteu reagent was used to determine the total phenolic content in the fruits and leaves of *L. australis* and *L. chinensis* extracts. The Folin–Ciocalteu reagent consists of complex polymeric ions (yellow solution) formed from phosphotungstic and phosphomolybdic heteropolyacids. This reagent produces complex molybdenum-tungsten blue from the oxidation of phenolates, which can be spectrophotometrically detected at 725 nm.^24^ As shown in Table 2, the order of the phenolic content was *L. australis* leaves > *L. chinensis* fruit > *L. australis* fruits > *L. chinensis* leaves with the values of 78.87, 44.50, 32.82 and 19.72 mg GAE g^−1^, respectively.

**Table 2:** Total phenolic and total flavonoid content of leaves and fruits extracts of *L. chinensis* and *L. australis*^a^

Samples	Total phenolic (mg GAE g^−1^)	Total flavonoid (mg RE g^−1^)
*L. chinensis* leaves	19.72 ± 3.49	4.82 ± 0.29
*L. chinensis* fruits	44.50 ± 1.60	17.75 ± 1.80
*L. australis* leaves	78.87 ± 0.29	8.16 ± 0.35
*L. australis* fruits	32.82 ± 0.77	12.81 ± 0.612

a Values are presented in mean ± SD (*n* = 3), expressed as mg gallic acid equivalent per g of dry sample (mg GAE g^−1^) for total phenolics and as mg rutin equivalent per g of dry sample (mg RE g^−1^) for total flavonoids.

Flavonoids are the widely distributed and most common group of plant phenolics, which are characterized by the presence of a benzo-γ-pyrone structure.^33^ The total flavonoids content was determined in the assigned extracts by reaction with sodium nitrite, followed by the formation of a colored complex of flavonoid-aluminum using aluminum chloride that was spectrophotometrically monitored at 510 nm.^24^ For both *Livistona* species, the samples containing the highest flavonoid content was *L. chinensis* fruit (17.75 mg RE g^−1^), followed by the *L. australis* fruit (12.81 mg RE g^−1^), *L. australis* leaves (8.16 mg RE g^−1^) and *L. chinensis* leaves (4.82 mg RE g^−1^), as indicated in Table 2.

The total flavonoid and phenolic contents for *L. chinensis* previously reported and the results were found to be 139.51 ± 5.90 μg g^−1^ dry plant and 112.07 ± 2.24 μg g^−1^ dry plant, respectively^28^ while the *L. speciosa* seed extract showed a total phenolic content of 2.35 mg of gallic acid/1 g of sample, and the total flavonoid was found 39.27 mg of rutin/1 g of sample.^34^ In our study, the *L. australis* leaves had the highest phenolic content, while the *L. chinensis* fruits showed a high flavonoid content.

### 3.3. Identification of the isolated compounds

**Fig. 2:**
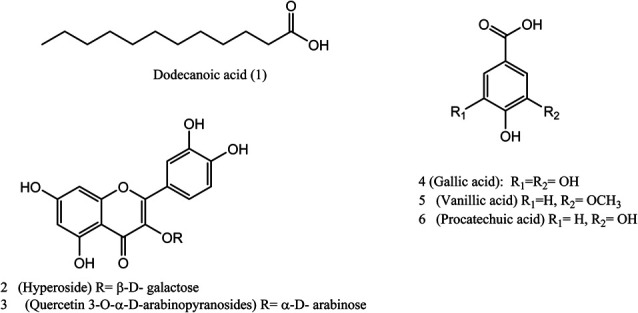
Structures of the isolated metabolites from the alcoholic extract of *L. australis* fruits.

Six known compounds were isolated and identified from the pericarp of *L. australis*: one fatty acid from the hexane fraction and five phenolic compounds from the ethyl acetate fraction. The chemical structures of the pure isolated compounds were elucidated using NMR spectrometry, and confirmed by comparing the obtained data with those previously reported in the literature (Fig. 2). The identified compounds were:

(1) dodecanoic acid; (lauric acid,C_12_H_24_O_2_),^35^ (2) hyperoside (quercetin 3-*O*-β-d-galactopyranoside, C_21_H_20_O_12_),^36^ (3) quercetin 3-*O*-α-d-arabinopyranoside (C_20_H_18_O_11_),^37^ (4) gallic acid (C_7_H_6_O_5_),^38^ (5) vanillic acid (C_8_H_8_O_4_) and (6) procatechuic acid (C_7_H_6_O_4_).^39^ These compounds have been isolated and characterized for the first time from *L. australis* fruits (spectroscopic data in ESI data, Fig. S9–S20†).

### 3.4. Biological studies

Phenolic compounds have been reported to lower the incidence of many chronic diseases, such as cancer, cardiovascular diseases (CVD), chronic respiratory diseases, diabetes and neurodegenerative diseases.^40^ In this work, extracts of *L. australis* showed a higher content of phenolic compounds than *L. chinensis*, so the biological evaluation was done for *L. australis* regarding anticholinesterase, and antiaging and antidiabetic properties.

#### 3.4.1. Anticholinesterase activity

A key neurotransmitter in the pathogenesis of Alzheimer's disease (AD) is acetylcholine, which is hydrolyzed by acetyl-cholinesterase (AChE) and butyryl-cholinesterase (BuChE). AD is associated with low level of acetylcholine in specific regions of the brain.^41,42^ AChE inhibitors, which led to high levels of acetylcholine in the brain, are considered to be the most effective strategy in AD treatment.^43,44^ Extracts of *L. australis* leaves and fruits were screened for AChE inhibitory activity using Ellman's spectrophotometric method. The results showed that the inhibitory concentration (IC_50_) was 65.55 ± 3.75 ng mL^−1^ and 122 ± 6.97 ng mL^−1^ for leaves and fruits, respectively, compared with donepezil as a standard with (IC_50_) at 49.44 ± 2.82 ng mL^−1^. The results (Table 3) revealed that the leaves extract was active as an antiacetylcholinesterase compared with the standard. Flavonoids are a promising class of natural compounds in the management of AD disease.^45^ A previous report on the anticholinesterase activity of *Sabal blackburniana* (Palmae) was performed, and the results showed a good activity of flavonoid aglycone against the acetylcholinesterase enzyme, such as luteolin, taxifolin, tricin, genistein, chrysoeriol and catechin.^46^*L. australis* leaves and fruit extracts contained many flavonoid aglycones, such as catechin, luteolin and tricin. Another report revealed that apigenin, vanillic acid and acacetin-7-*O*-β-d-glucoside showed potent anticholinesterase activity.^26^ Finally, it can be concluded that the anti-cholinesterase activity may refer to flavonoids and phenolic acids detected in the analyzed extracts.

**Table 3:** Anticholinesterase, telomerase activation, and DPP-IV inhibitory activities of alcoholic extract from the leaves and fruits of *L. australis*

Extract/control	Anticholinesterase IC_50_ (ng ml^−1^)	Telomerase activation conc (ng ml^−1^)	DPP_4_ inhibition IC_50_ (ng ml^−1^)
*L. australis* leaves	65.55 ± 3.75	12.23 ± 0.59 (1.49)	90.8 ± 4.48
*L. australis* fruits	122 ± 6.97	11.5 ± 0.65 (1.41)	359 ± 17.7
Donepezil	49.44 ± 2.82	—	—
Curcumin	—	12.67 ± 0.49 (1.54)	—
Sitagliptin	—	—	63.8 ± 3.15
Control	—	8.206 ± 0.33 (1)	—

#### 3.4.2. Antiaging activity

Telomere damage is one of the primary causes of aging. It is noteworthy that many chronic diseases, such as cancer, heart diseases, diabetes, and kidney diseases, are related to ageing.^7^ In human cells, telomerase expression and activity decline with ageing.^47^ Gene therapy with hTERT or telomerase activators are the strategies commonly used for treatment of telomerase-dependent disease. However, gene therapy is not a practical medical intervention, so the attractive alternative would be a chemical telomerase activator, which would allow for a more precise control over the dose and timing.^48^ Previous studies on the root extract of *Astragalus membranaceus* led to an increase in the concentration of telomerase enzyme in the tested cells.^49^ Our results (Table 3) revealed that *L. australis* leaves were a potent extract as it caused a 1.49-fold increase in the telomerase activity, followed by *L. australis* fruit extract with a 1.41-fold increase in the telomerase activity. Meanwhile, curcumin as the standard caused a 1.54-fold increase in telomerase activity.

#### 3.4.3. Antidiabetic activity

DPP-IV is a serine protease that cleaves N-terminal dipeptides from polypeptides. It regulates the bioactivity of many peptides, including the glucagon-like peptide-1 (GLP-1) and glucose-dependent insulinotropic polypeptide (GIP).^50^ DPP-IV inhibitors are crucial for the management of type II diabetes. This study reports for the first time the DPP-IV inhibition potential of *L. australis* leaves and fruits extracts as an antidiabetic, and compared it with the control drug sitagliptin. The results (Table 3) showed that the leaves extract demonstrated the most significant action with an IC_50_ value of 90.8 ± 4.48 ng mL^−1^ compared to the control IC_50_ = 63.8 ± 3.15 ng mL^−1^. Previous reports on the antidiabetic potential of *L. chinensis* was evaluated by measuring the α-glucosidase inhibitory effect and protein tyrosine phosphatase 1B (PTP1B) inhibitory effect, and the results showed that the butyl alcohol fraction was the most potent as an α-glucosidase and PTP1B inhibitor.^21^ The activity of the leaf extract may be attributed to the flavonoid content of the extract. Many flavonoids reported as DPP-IV inhibitors, such as quercetin, luteolin, apigenin, kaempferol, flavone, hesperetin, naringenin and genistin, in addition to glycosides as isoquercetin, kaempferol 7-*O*-α-l-rhamnoside, vitexin, rutin, isorhamnetin-3-*O*-glucoside and isorhamnetin-3-*O*-rutinoside, have also been reported as DPP-IV inhibitors.^8,51,52^

## 4. Conclusion

This research focused on the phytochemical characterization of the leaves and fruits of *Livistona chinensis* and *L. australis*, and the biological activities of *L. australis* leaves and fruits. The extracts were rich with phenolic compounds, especially flavonoids. Biological evaluation showed that the leaves extract displayed the best inhibitory effects against acetyl cholinesterase and DPP-IV, and good potentiation for telomerase activity. However, further experimental studies, such as *in vivo* animal studies and *in silico* study, could be planned depending on the present results.

## Ethical statement

Ethics approval was not required for this research.

## Author contributions

Collection, drying, and extraction of the plant: S. O. A., LC/MS analysis: U. R. A., compound isolation and identification: A. E., conceptualization, and methodology: S. S. E. and A. I. O., all authors contributed to the writing, reviewing, revising and editing of the manuscript.

## Conflicts of interest

The authors declare that there is no conflict of interest.

## Supplementary Material

RA-013-D3RA01229H-s001

## References

[cit1] Yiannopoulou K. G., Papageorgiou S. G. (2020). J. Cent. Nerv. Syst. Dis..

[cit2] Tamfu A. N., Kucukaydin S., Yeskaliyeva B., Ozturk M., Dinica R. M. (2021). Molecules.

[cit3] Blackburn E. H., Greider C. W., Szostak J. W. (2006). Nat. Med..

[cit4] Harley C. B. (2005). Curr. Mol. Med..

[cit5] Lulkiewicz M., Bajsert J., Kopczynski P., Barczak W., Rubis B. (2020). Mol. Biol. Rep..

[cit6] Aydin Y. , in Molecular Basis and Emerging Strategies for Anti-aging Interventions, ed. S. I. Rizvi and U. Çakatay, Springer Singapore, Singapore, 2018, pp. 97–109, DOI: 10.1007/978-981-13-1699-9_7

[cit7] López-Otín C., Blasco M. A., Partridge L., Serrano M., Kroemer G. (2013). Cell.

[cit8] Gao F., Fu Y., Yi J., Gao A., Jia Y., Cai S. (2020). J. Agric. Food Chem..

[cit9] Kaur J., Singla R., Jaitak V. (2018). Lett. Drug Des. Discovery.

[cit10] Wu T., Rayner C. K., Horowitz M. (2015). Metab. Control.

[cit11] Langley A. K., Suffoletta T. J., Jennings H. R. (2007). Pharmacotherapy.

[cit12] Chen P., Yang J. (2007). Chin. Tradit. Herb Drugs.

[cit13] Yuan T., Yang S.-P., Zhang H.-Y., Liao S.-G., Wang W., Wu Y., Tang X.-C., Yue J.-M. (2009). J. Asian Nat. Prod. Res..

[cit14] Zeng X., Qiu Q., Jiang C., Jing Y., Qiu G., He X. (2011). Fitoterapia.

[cit15] El-Desouky S. K., Kassem M. E., Fifi Z. I. A., El-Deen A. M. G. (2009). Nat. Prod. Commun..

[cit16] Kassem M. E., Shoela S., Marzouk M. M., Sleem A. A. (2012). Nat. Prod. Res..

[cit17] Lin W., Zhao J., Cao Z., Zhuang Q., Zheng L., Cai Q., Chen D., Wang L., Hong Z., Peng J. (2013). Oncol. Rep..

[cit18] Li S., Luo S., Chen H., Zheng Y., Lin L., Yao H., Lin X. (2019). Drug Des., Dev. Ther..

[cit19] Ibrahim H., Elshaarawy F., Haggag E. (2018). J. Adv. Pharm. Educ. Res..

[cit20] El-Hawary S. S., Ali T. F., El-Ela S. O. A., Elwekeel A., Abdelmohsen U. R., Owis A. I. (2022). RSC Adv..

[cit21] Wang Y., Zhai J., Yang D., Han N., Liu Z., Liu Z., Li S., Yin J. (2021). Oxid. Med. Cell. Longevity.

[cit22] El-Hawary S. S., Owis A. I., El-Ela S.
O. A., Elwekeel A. (2022). Egypt. J. Chem..

[cit23] Owis A. I., El-Hawary M. S., El Amir D., Aly O. M., Abdelmohsen U. R., Kamel M. S. (2020). RSC Adv..

[cit24] Bakar M. F. A., Mohamed M., Rahmat A., Fry J. (2009). Food Chem..

[cit25] Dhanasekaran S., Perumal P., Palayan M. (2015). J. Appl. Pharm. Sci..

[cit26] Refaey M. S., Abdelhamid R. A., Elimam H., Elshaier Y. A., Ali A., Orabi M. A. (2021). Bioorg. Chem..

[cit27] Kempegowda P. K., Zameer F., Narasimashetty C. K., Kollur S. P., Murari S. K. (2018). Afr. J. Tradit., Complementary Altern. Med..

[cit28] Ahmed R., Elkhrisy E., EL-kashak W. A. H., El Raey M., Nassar M., Aboutabl E.-S. A. (2019). J. Adv. Pharm. Educ. Res..

[cit29] Zeng X., Wang Y., Qiu Q., Jiang C., Jing Y., Qiu G., He X. (2012). Fitoterapia.

[cit30] Wu M., Wang C., Mai C., Chen J., Lai X., He L., Huang S., Zhang X. (2019). J. Funct. Foods.

[cit31] Olennikov D., Zilfikarov I., Khodakova S. (2013). Chem. Nat. Compd..

[cit32] S Elshaarawy F., A Mina S., M Gabr N., M Abdelkhalik S., Kamel R., A Ibrahim H., Haggag E. G. (2018). J. Adv. Pharm. Educ. Res..

[cit33] Mabry T. , Markham K. R. and Thomas M. B., The systematic identification of flavonoids, Springer Science & Business Media, 2012

[cit34] Takolpuckdee P. (2016). Thai J. Pharm. Sci..

[cit35] El-ghaly E. S. (2014). Al-Azhar J. Pharm. Sci..

[cit36] Xiao Z., Wu H., Wu T., Shi H., Hang B. (2006). Chem. Nat. Compd..

[cit37] De Almeida A., Miranda M., Simoni I., Wigg M., Lagrota M., Costa S. (1998). Phytother. Res..

[cit38] Lim Y. A., Mei M. C., Kusumoto I. T., Miyashiro H., Hattori M., Gupta M. P., Correa M. J. P. R. (1997). Phytother. Res..

[cit39] Yu Y., Gao H., Tang Z., Song X., Wu L. J. (2006). Asian J. Tradit. Med..

[cit40] Haminiuk C. W., Maciel G. M., Plata-Oviedo M. S., Peralta R. M. (2012). Int. J. Food Sci. Technol..

[cit41] Zarotsky V., Sramek J. J., Cutler N. R. (2003). Am. J. Health-Syst. Pharm..

[cit42] Sun J., Wang B., Niu Y., Tan Y., Fan C., Zhang N., Xue J., Wei J., Xiang J. (2020). Entropy.

[cit43] Arnold S. E., Kumar A. (1993). Med. Clin. North Am..

[cit44] Schneider J. A., Arvanitakis Z., Bang W., Bennett D. A. (2007). Neurology.

[cit45] Bakoyiannis I., Daskalopoulou A., Pergialiotis V., Perrea D. (2019). Biomed. Pharmacother..

[cit46] El-Hawwary S. S., Abd Almaksoud H. M., Saber F. R., Elimam H., Sayed A. M., El Raey M. A., Abdelmohsen U. R. (2021). RSC Adv..

[cit47] Cong Y.-S., Wright W. E., Shay J. W. (2002). Microbiol. Mol. Biol. Rev..

[cit48] Molgora B., Bateman R., Sweeney G., Finger D., Dimler T., Effros R. B., Valenzuela H. F. (2013). Cells.

[cit49] Harley C. B., Liu W., Blasco M., Vera E., Andrews W. H., Briggs L. A., Raffaele J. M. (2011). Rejuvenation Res..

[cit50] Fujiwara K., Inoue T., Yorifuji N., Iguchi M., Sakanaka T., Narabayashi K., Kakimoto K., Nouda S., Okada T., Ishida K. (2015). J. Clin. Biochem. Nutr..

[cit51] Shaikh S., Lee E.-J., Ahmad K., Ahmad S.-S., Lim J.-H., Choi I. (2021). Pharmaceuticals.

[cit52] Zhao B. T., Le D. D., Nguyen P. H., Ali M. Y., Choi J.-S., Min B. S., Shin H. M., Rhee H. I., Woo M. H. (2016). Chem.-Biol. Interact..

