# Supplementary material for: Metabolic profiling and biological activity of two Livistona species: L. chinensis and L. australis
Source: RSC Adv. 2023 May 15;13(22):14855–62. doi: 10.1039/d3ra01229h (PMC10184005; doi:10.1039/d3ra01229h)
Supplement: RA-013-D3RA01229H-s001 [file RA-013-D3RA01229H-s001.pdf]

Electronic Supplementary Material (ESI) for RSC Advances.
This journal is © The Royal Society of Chemistry 2023

Supplementary Data

Metabolic profiling and biological activity of two *Livistona* species: *L. chinensis* and *L. australis*

Seham S. El-hawary[1#], Ahlam Elwekeel[2*#], Sara O. Abo El-Ela[2], Usama Ramadan Abdelmohsen[3,4], and Asmaa I. Owis[2,5]

[1]Department of Pharmacognosy, Faculty of pharmacy, Cairo University, Cairo, Egypt

[2]Department of Pharmacognosy, Faculty of Pharmacy, Beni-Suef University, Beni-Suef, Egypt

[3]Department of Pharmacognosy, Faculty of Pharmacy, Minia University, Minia, Egypt

[4]Department of Pharmacognosy, Faculty of Pharmacy, Deraya University, New Minia, Egypt

[5]Department of Pharmacognosy, Faculty of Pharmacy, Heliopolis University for Sustainable Development, Cairo, Egypt

[#] These authors contributed equally to this work

*Corresponding author

Ahlam Elwekeel

E-mail: **Ahlam_hasham2000@yahoo.com**

Ahlam.hassanain@pharm.bsu.edu.eg

List of figures

Figure S1: LC-MS chromatogram of the *Livistona australis* leaves (Positive ion mode)
Figure S2: LC-MS chromatogram of the *Livistona australis* leaves (negative ion mode)
Figure S3: LC-MS chromatogram of the *Livistona australis* fruits (Positive ion mode)
Figure S4: LC-MS chromatogram of the *Livistona australis* fruits (negative ion mode)
Figure S5: LC-MS chromatogram of the *Livistona chinensis* leaves (Positive ion mode**)**
Figure S6: LC-MS chromatogram of the *Livistona chinensis* leaves (negative ion mode)
Figure S7: LC-MS chromatogram of the *Livistona chinensis* fruits (Positive ion mode).
Figure S8: LC-MS chromatogram of the *Livistona chinensis* fruits (negative ion mode)
Figure S9: ^{1}H-NMR spectrum of dodecanoic acid ($CDCl_3$)
Figure S10: DEPT-Q NMR spectrum of dodecanoic acid (DMSO)
Figure S11: ^{1}H-NMR spectrum of hyperoside (CD_3OD)
Figure S12: DEPT-Q NMR spectrum of hyperoside (CD_3OD)
Figure S13: ^{1}H-NMR spectrum of quercetin 3-*O*-α-D-arabinopyranoside (CD_3OD)
Figure S14: DEPT-Q NMR spectrum of quercetin 3-*O*-α-D-arabinopyranoside (CD_3OD)
Figure S15: ^{1}H-NMR spectrum of gallic acid (CD_3OD)
Figure S16: DEPT-Q NMR spectrum of gallic acid (CD_3OD)
Figure S17: ^{1}H-NMR spectrum of vanillic acid (CD_3OD)
Figure S18: DEPT-Q NMR spectrum of vanillic acid (CD_3OD)
Figure S19: ^{1}H-NMR spectrum of procatechuic acid (CD_3OD)
Figure S20: DEPT-Q NMR spectrum of procatechuic acid (CD_3OD)

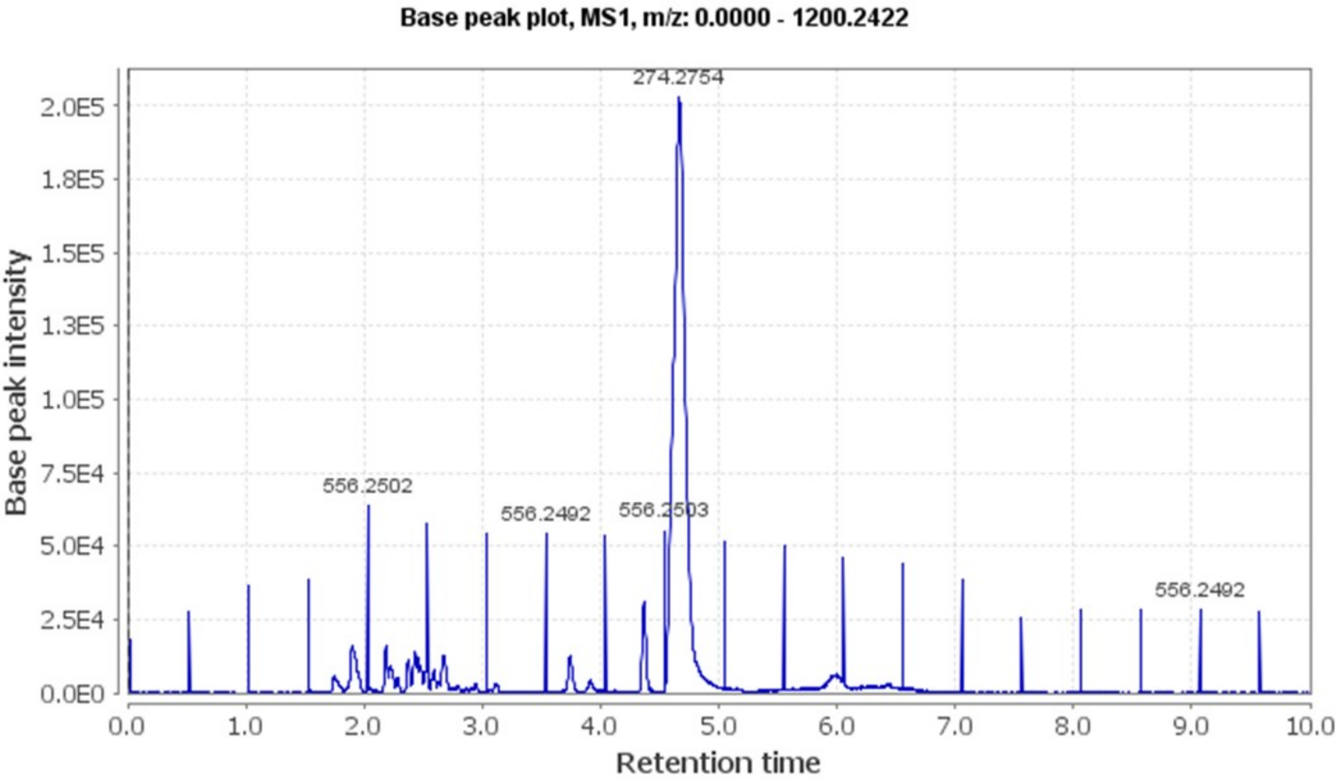

Figure S1: LC-MS chromatogram of the *Livistona australis* leaves (Positive ion mode)

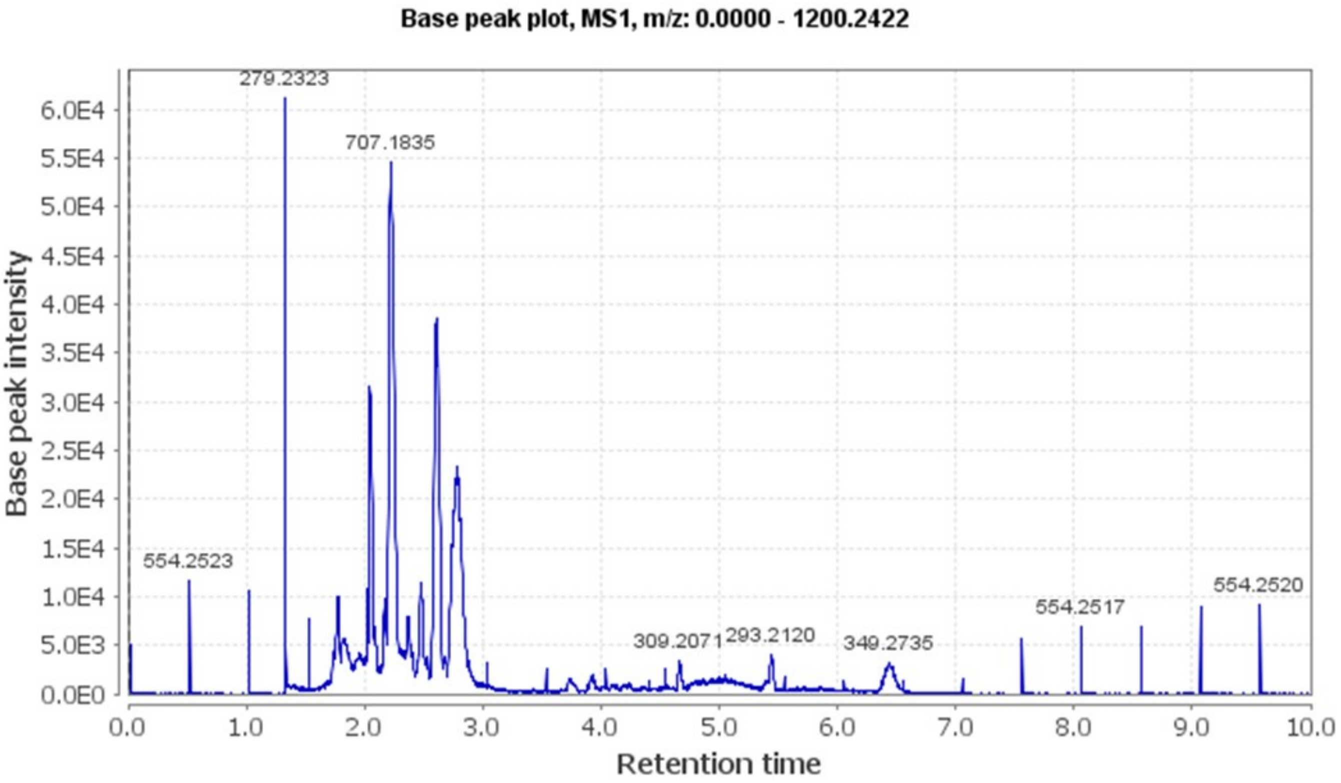

Figure S2: LC-MS chromatogram of the *Livistona australis* leaves (negative ion mode)

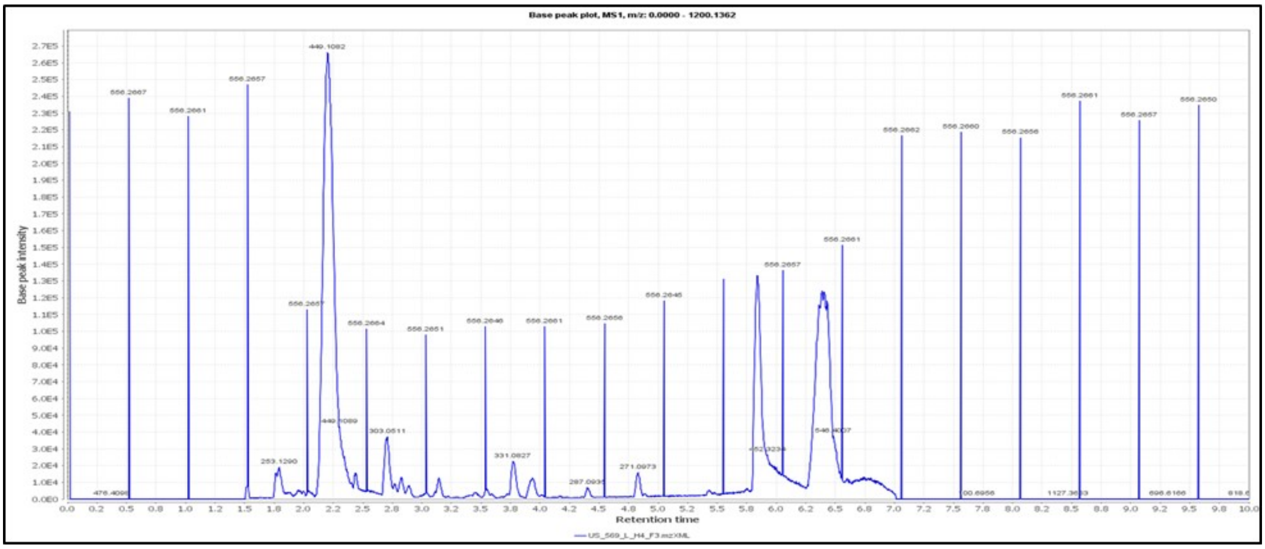

Figure S3: LC-MS chromatogram of the *Livistona australis* fruits (Positive ion mode)

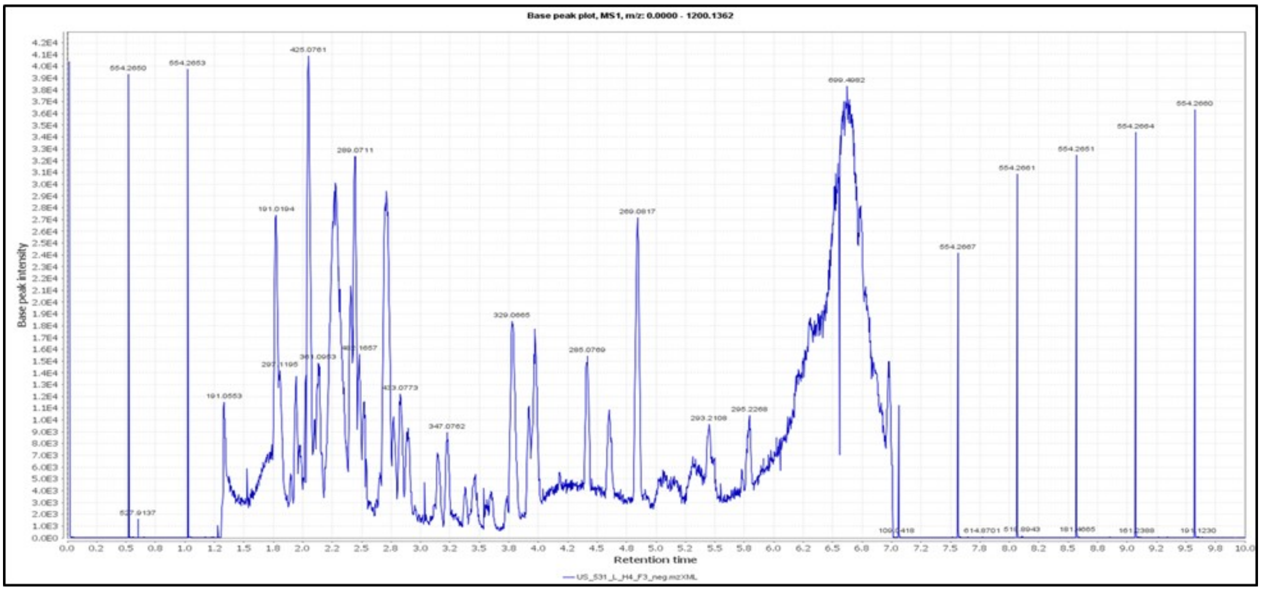

Figure S4: LC-MS chromatogram of the *Livistona australis* fruits (negative ion mode)

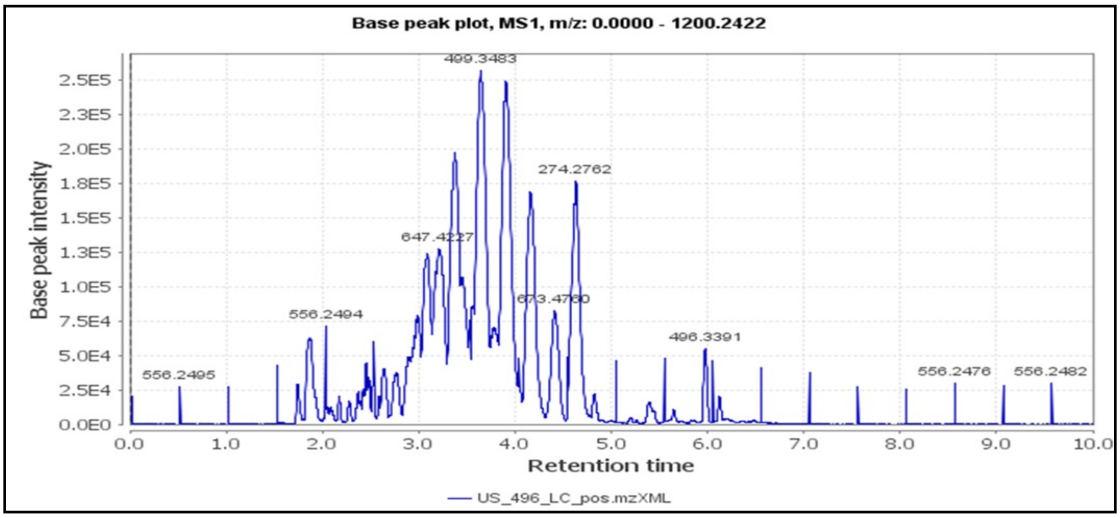

Figure S5: LC-MS chromatogram of the *Livistona chinensis* leaves (Positive ion mode)

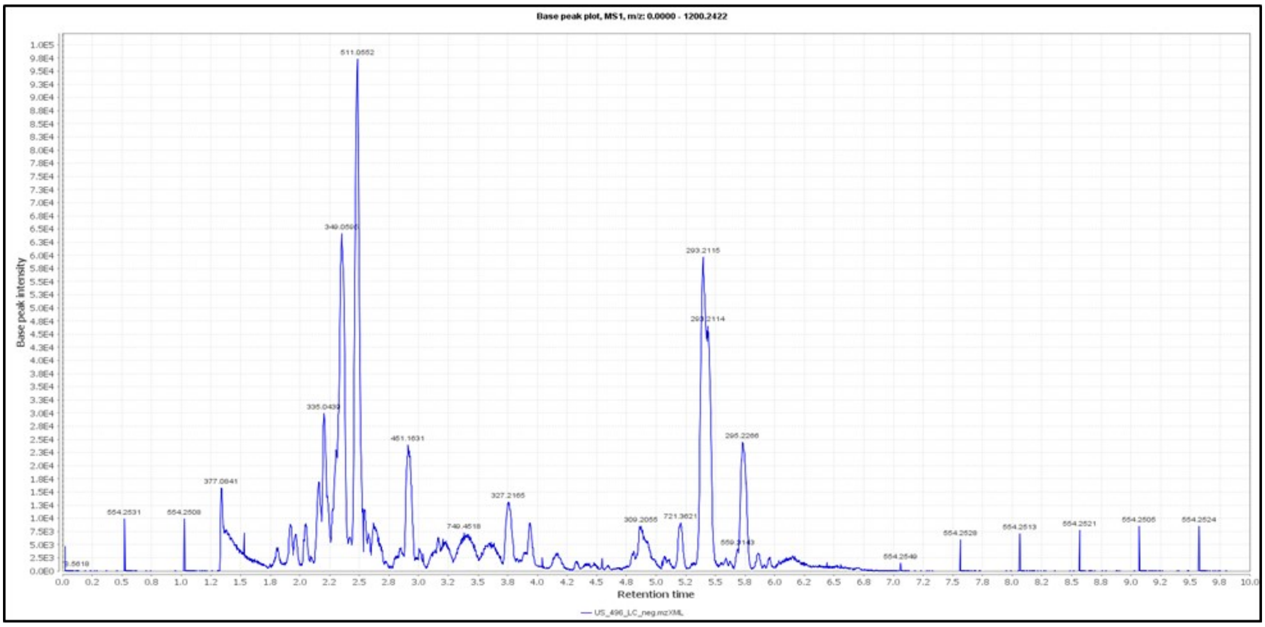

Figure S6: LC-MS chromatogram of the *Livistona chinensis* leaves (negative ion mode)

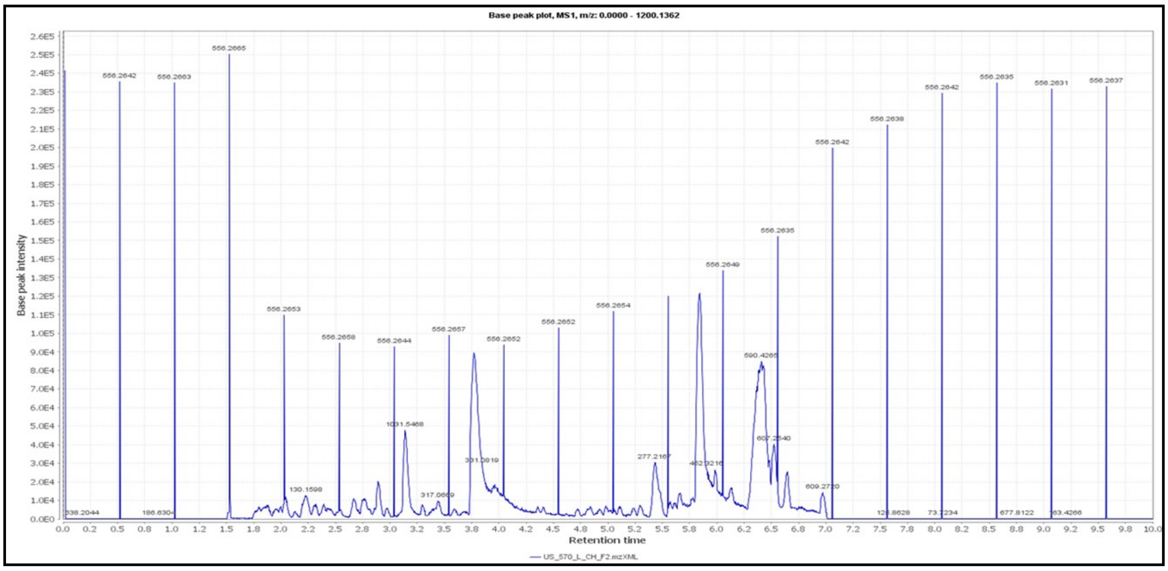

Figure S7: LC-MS chromatogram of the *Livistona chinensis* fruits (Positive ion mode).

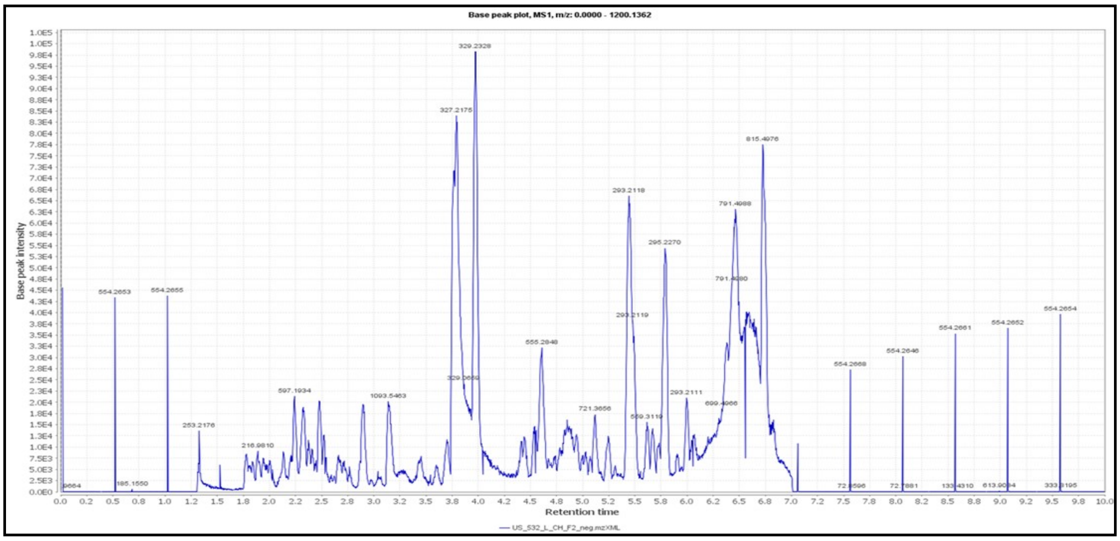

Figure S8: LC-MS chromatogram of the *Livistona chinensis* fruits (negative ion mode)

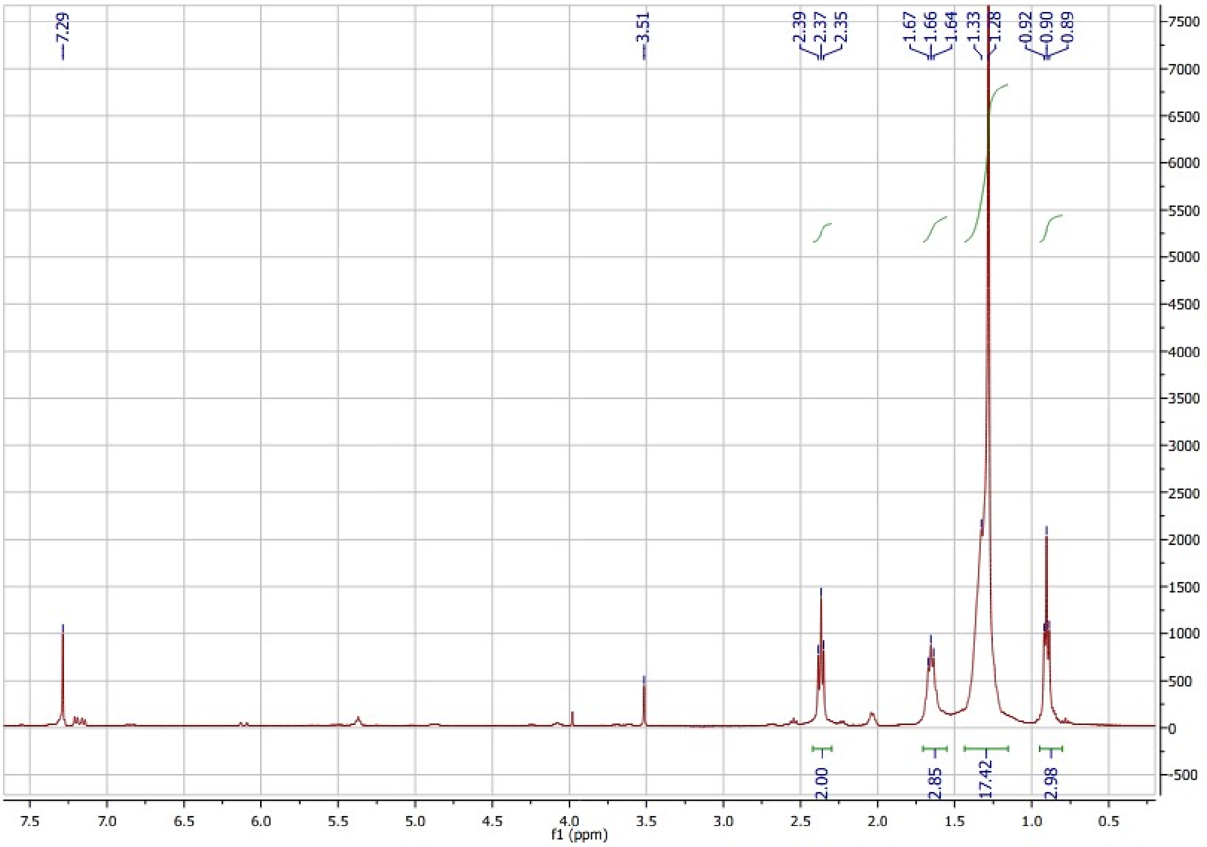

Figure S9: ^{1}H-NMR spectrum of dodecanoic acid, $C_{12}H_{24}O_2$ ($CDCl_3$)

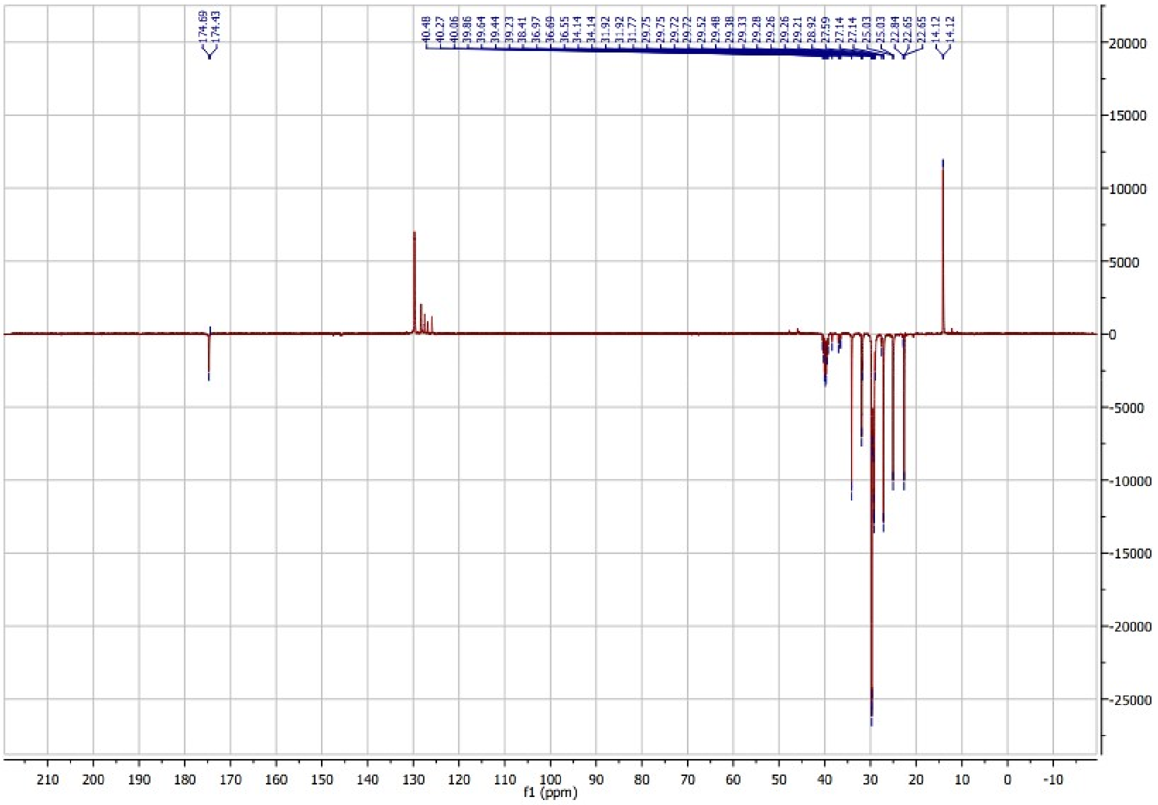

Figure S10: DEPT-Q NMR spectrum of dodecanoic acid; $C_{12}H_{24}O_2$ (DMSO)

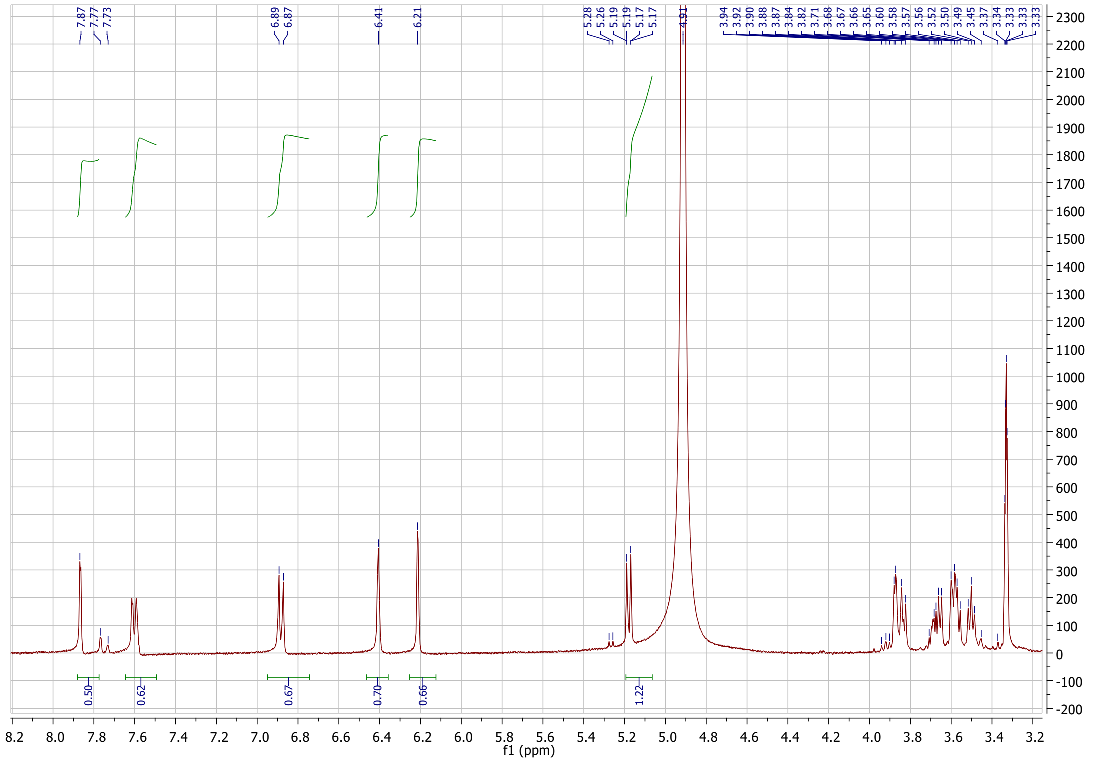

Figure S11: ^{1}H-NMR spectrum of hyperoside, $C_{21}H_{20}O_{12}$ (CD_3OD)

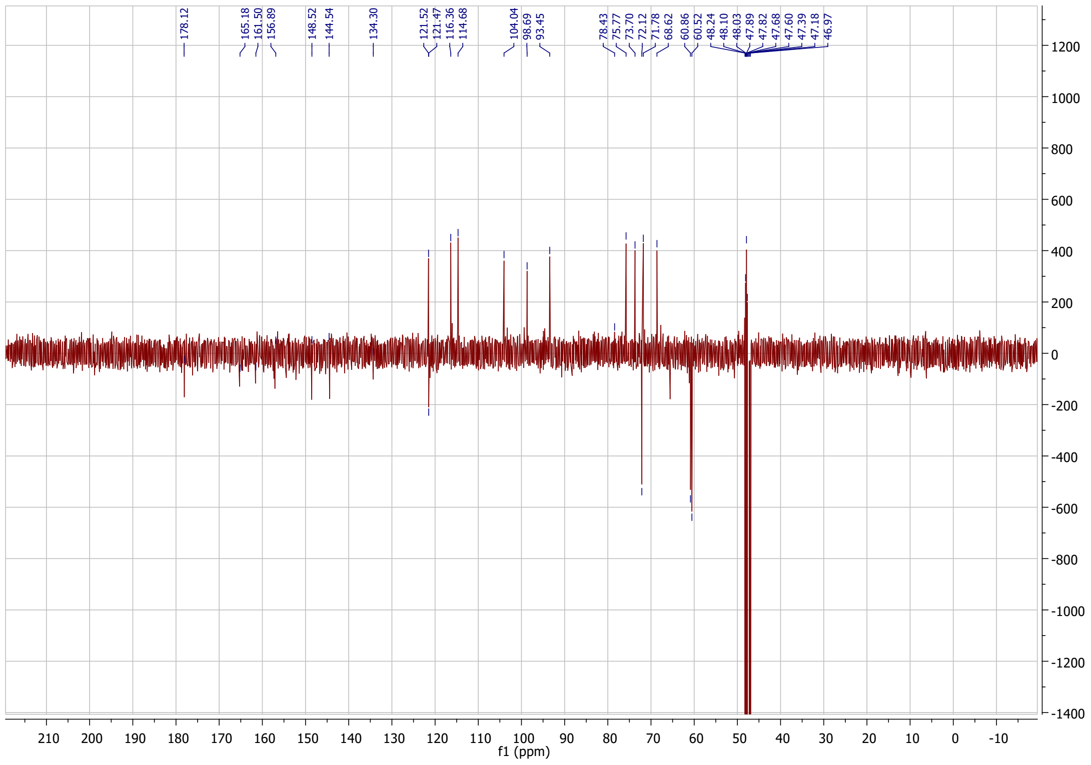

Figure S12: DEPT-Q NMR spectrum of hyperoside; $C_{21}H_{20}O_{12}$ (CD_3OD)

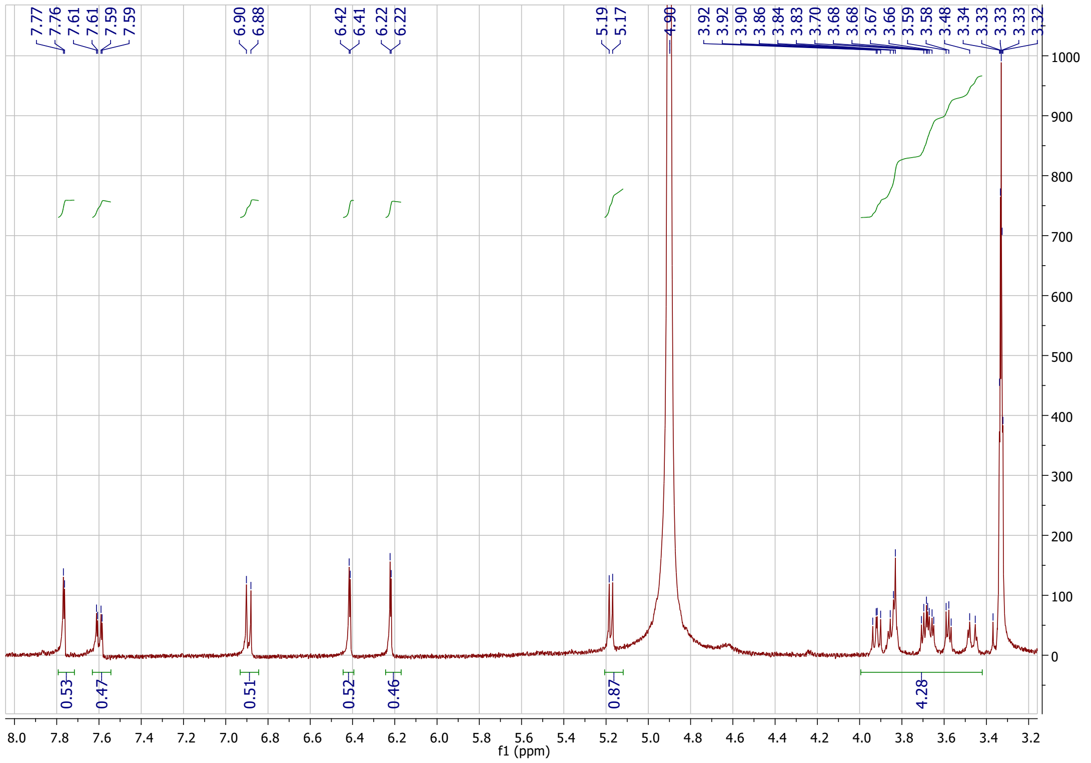

Figure S13: ^{1}H-NMR spectrum of quercetin 3-*O*-α-D-arabinopyranoside; $C_{20}H_{18}O_{11}$ (CD_3OD)

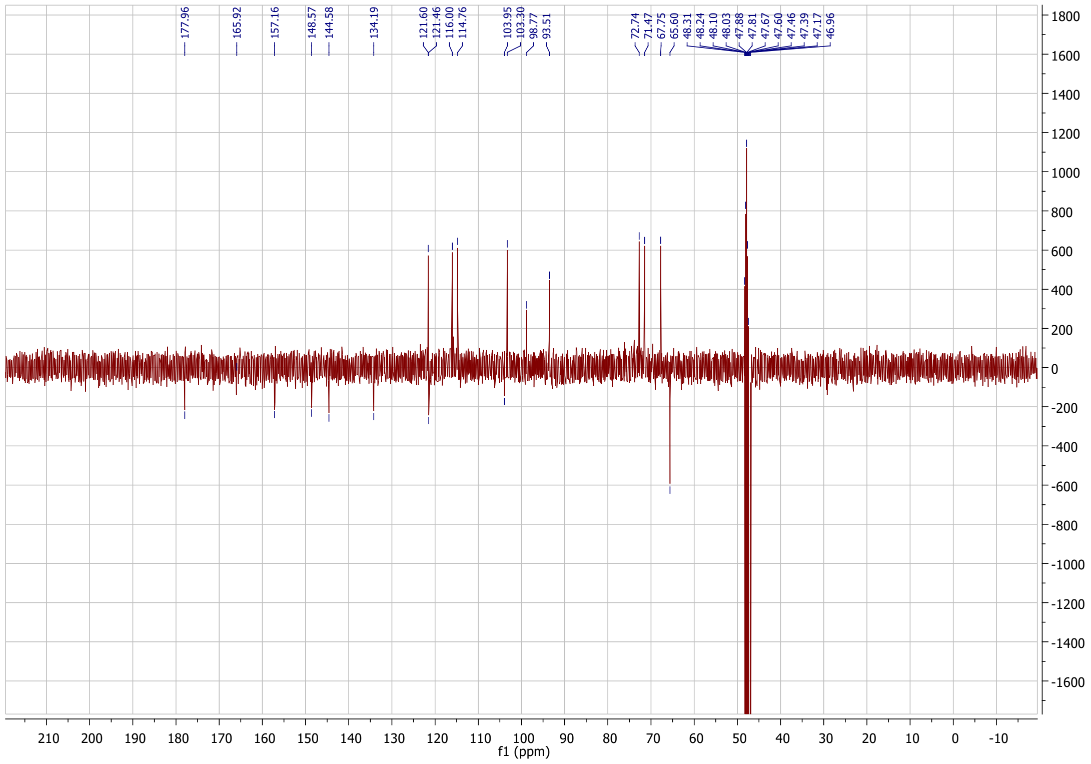

Figure S14: DEPT-Q NMR spectrum of quercetin 3-*O*-α-D-arabinopyranoside; $C_{20}H_{18}O_{11}$ (CD_3OD)

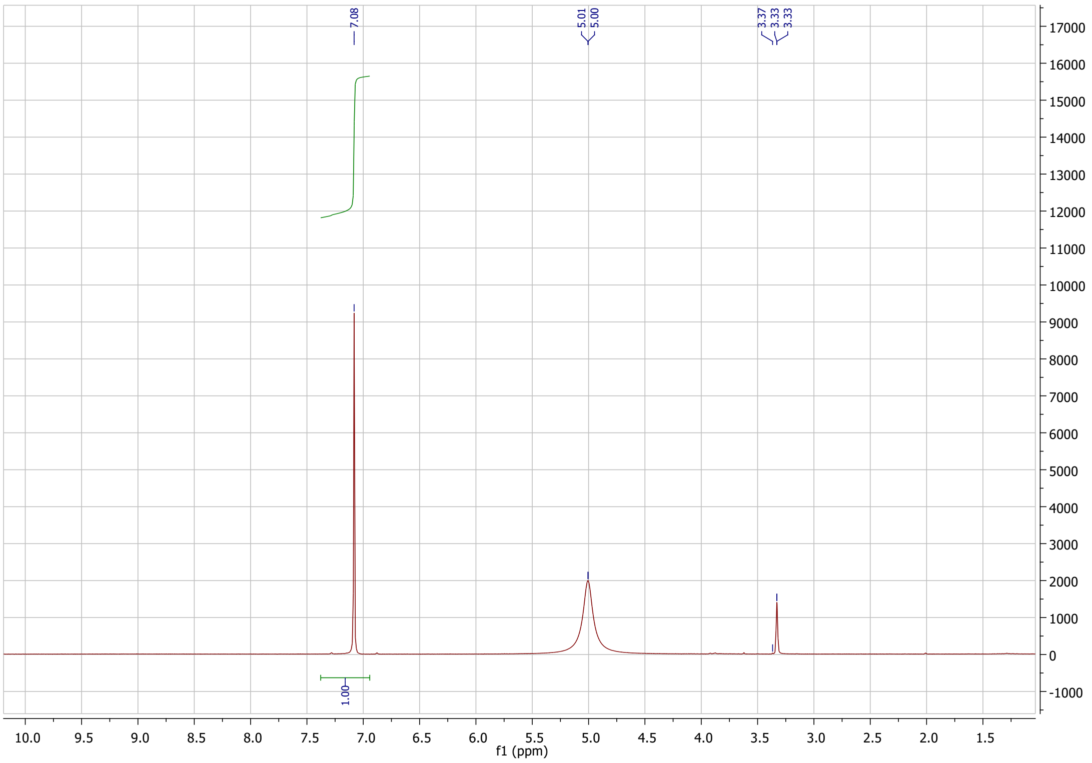

Figure S15: ^{1}H-NMR spectrum of gallic acid; $C_7H_6O_5$ (CD_3OD)

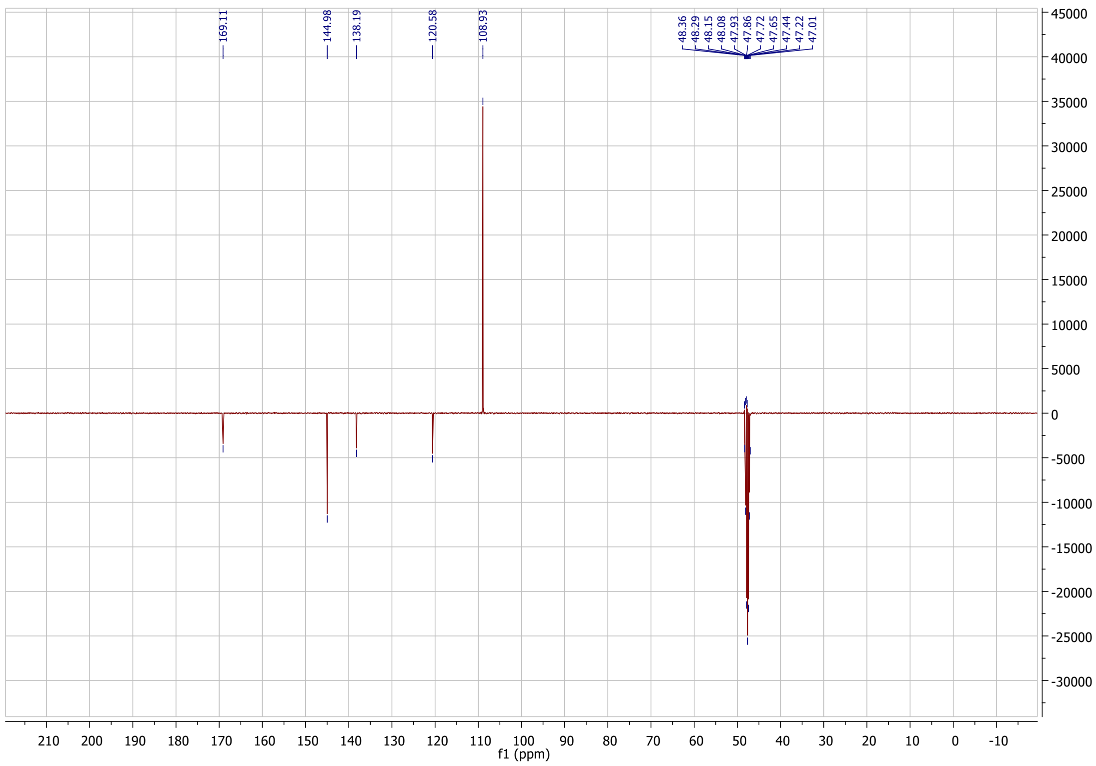

Figure S16: DEPT-Q NMR spectrum of gallic acid; $C_7H_6O_5$ (CD_3OD)

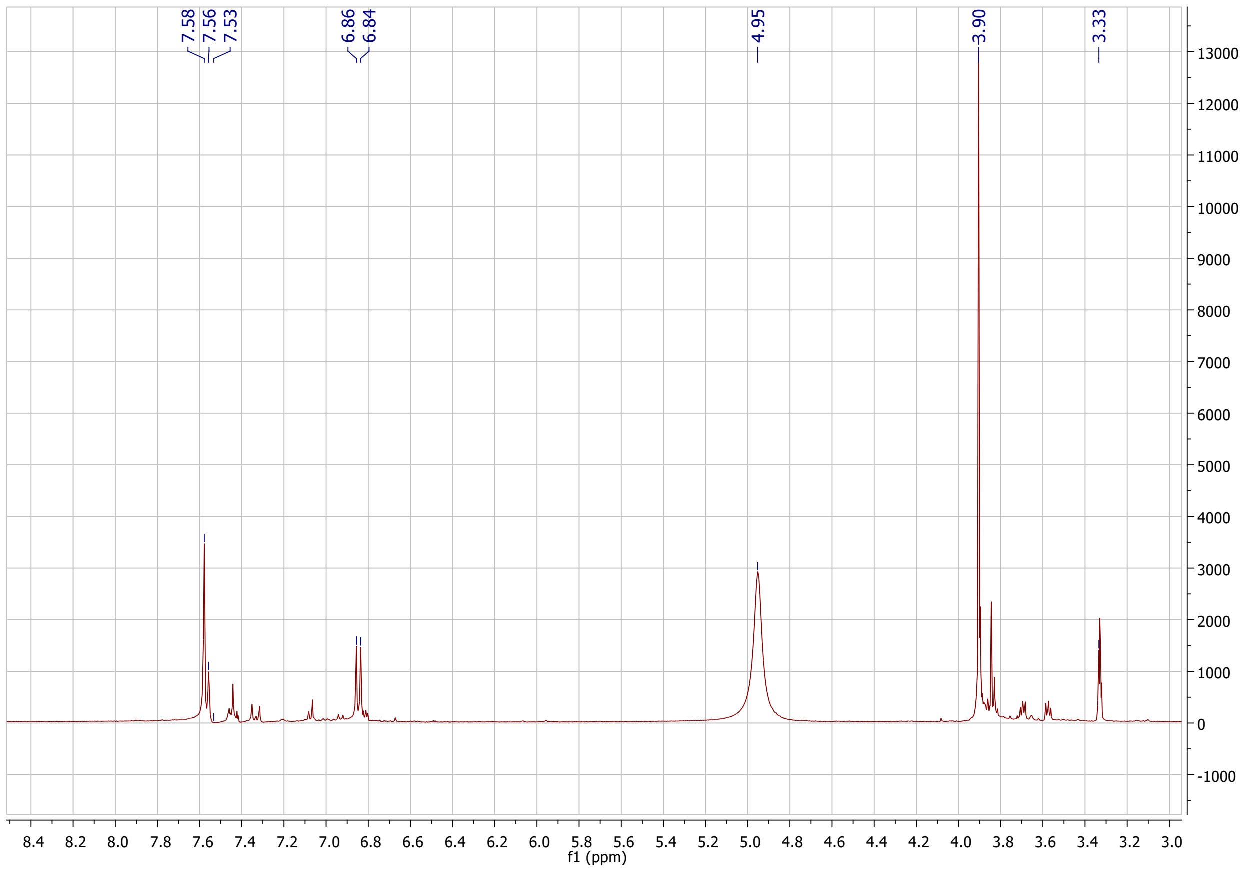

Figure S17: ^{1}H-NMR spectrum of vanillic acid; $C_8H_8O_4$ (CD_3OD)

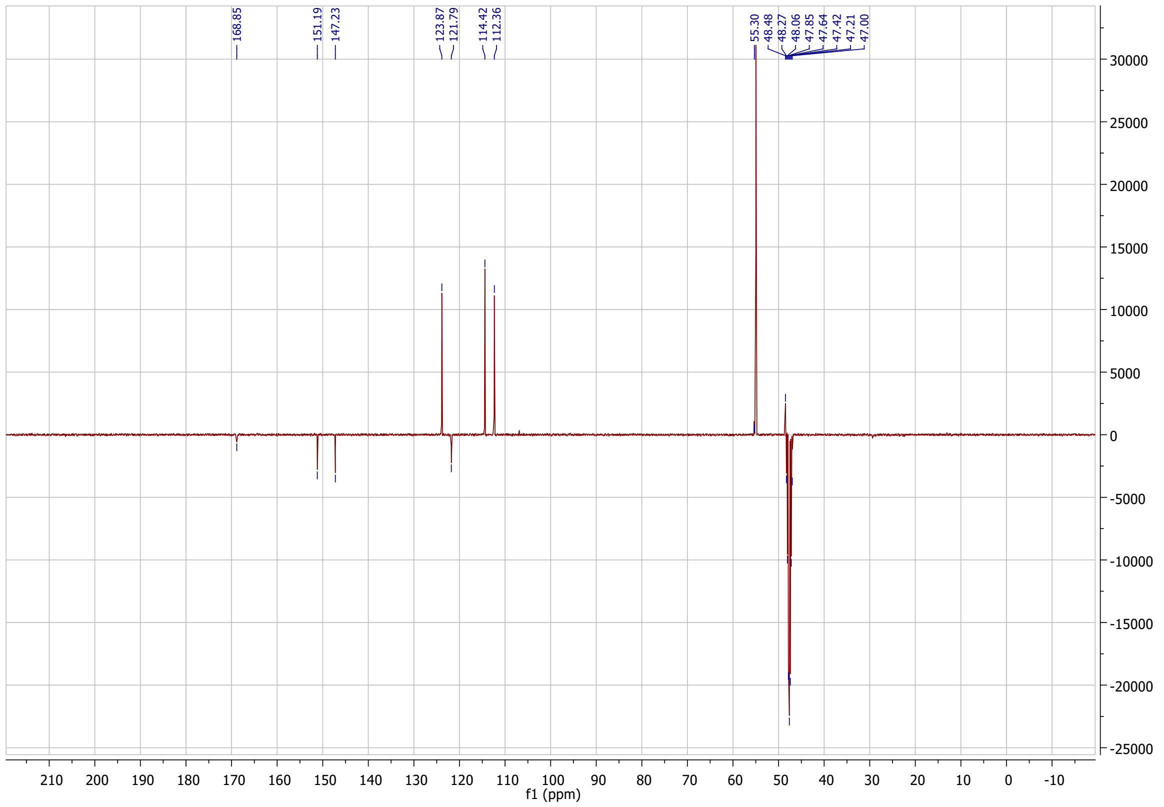

Figure S18: DEPT-Q NMR spectrum of vanillic acid; $C_8H_8O_4$ (CD_3OD)

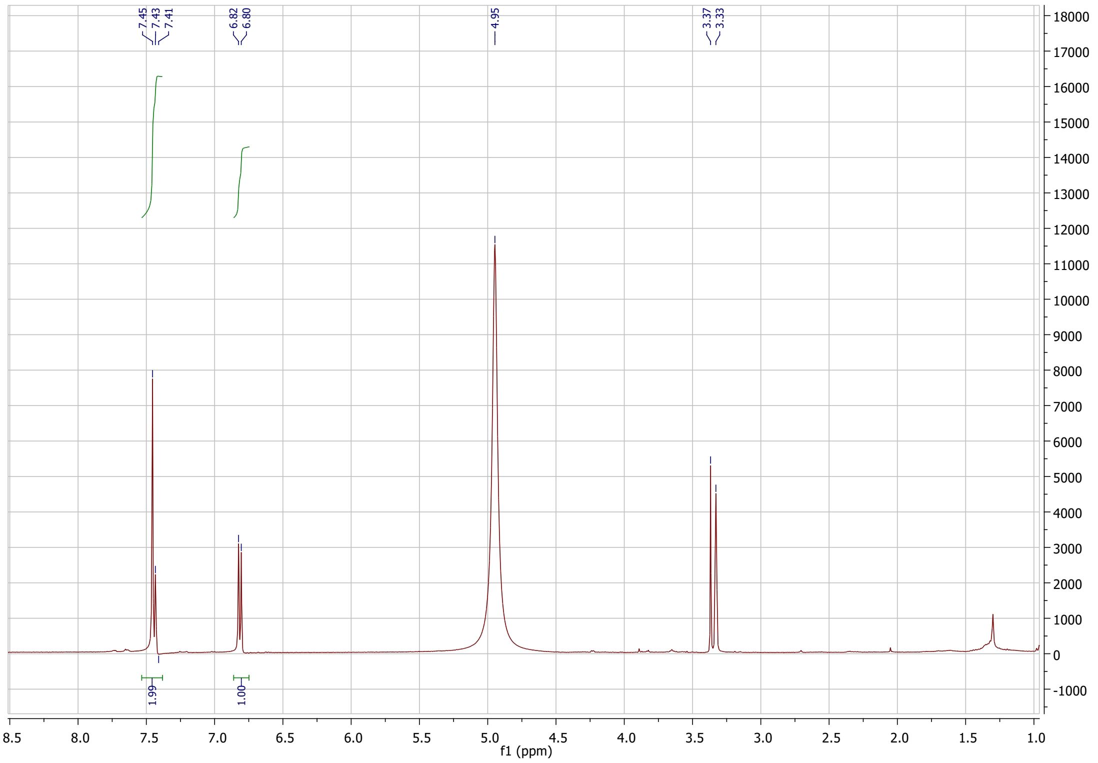

Figure S19: ^{1}H-NMR spectrum of procatechuic acid; $C_7H_6O_4$ (CD_3OD)

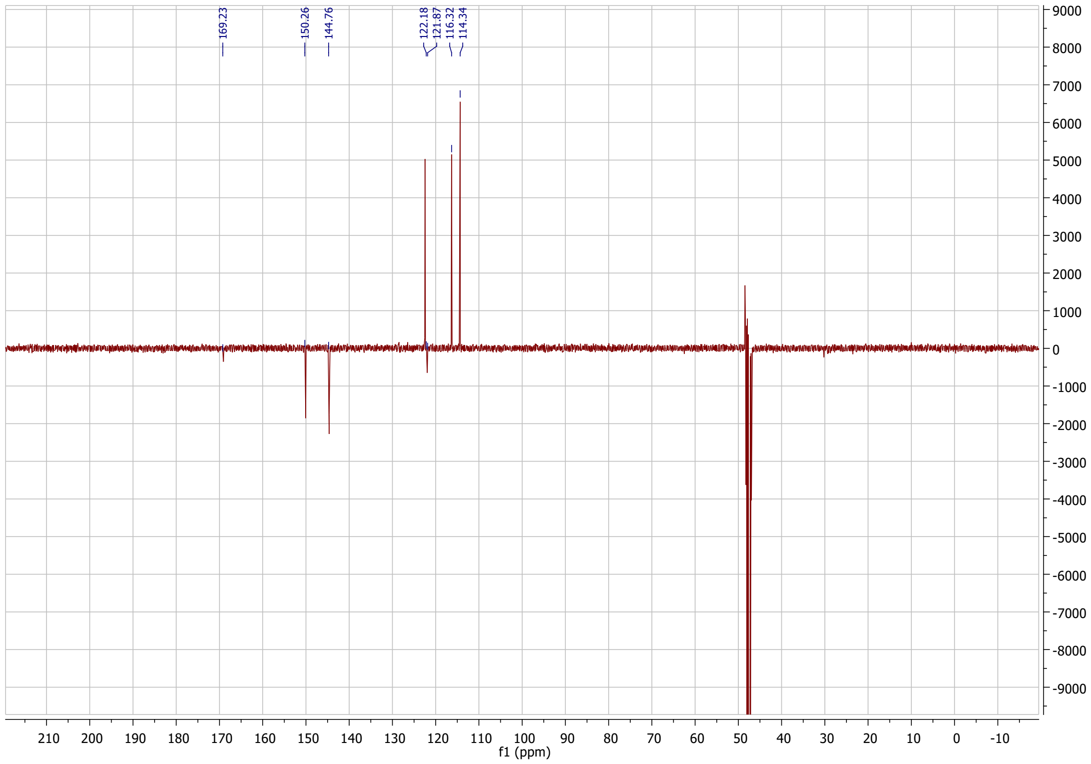

Figure S20: DEPT-Q NMR spectrum of procatechuic acid; $C_7H_6O_4$ (CD_3OD)
